# Supplementary material for: The meaning of caring for patients with cancer among traditional medicine practitioners in Uganda: A grounded theory approach
Source: PLOS Glob Public Health. 2023 Jul 17;3(7):e0001764. doi: 10.1371/journal.pgph.0001764 (PMC10351711; doi:10.1371/journal.pgph.0001764)
Supplement: S1 Checklist — (DOCX) [file pgph.0001764.s001.docx]

Inclusivity in global research

PLOS’ policy on inclusivity in global research aims to improve transparency in the reporting of research performed outside of researchers’ own country or community and ensures that PLOS publications reporting global research adhere to high standards for research ethics and authorship. Authors of relevant research articles may be asked to complete the questionnaire below, which outlines ethical, cultural, and scientific considerations specific to inclusivity in global research. This questionnaire may be requested when researchers have travelled to a different country to conduct research, if research uses samples collected in another country, research with Indigenous populations or their lands, or if research is on cultural artefacts. Researchers travelling to another country solely to use laboratory equipment will not normally be required to complete the questionnaire. However, the questionnaire can be requested at the journal’s discretion for any submission – if you have been requested to complete this questionnaire by the PLOS journal you submitted to, please do so.

Please complete the questionnaire below and include this as a Supporting Information file with your manuscript. Note that if your paper is accepted for publication, this checklist will be published with your article in the supporting information files. Please ensure that you reference the checklist in the main body of your manuscript. We suggest adding a subsection ‘Inclusivity in global research’ to your Methods section and adding the following sentence: “Additional information regarding the ethical, cultural, and scientific considerations specific to inclusivity in global research is included in the Supporting Information (SX Checklist)”

The questions have been designed to be applicable to a wide range of study types, and there are subsections for both human subjects research and non-human subjects research. If any of the questions are not relevant to your research please mark them as “N/A” as appropriate.

**Ethical considerations, permits and authorship**

*This section is applicable to all research types.*

Provide details as to who granted permissions and/or consent for the study to take place in the Methods section of your manuscript. This should include the names of **all** ethics boards, governmental organizations, community leaders or other bodies that provided approval for the study. If individuals provided approval refer to these people by their role or title but do not list their name(s).

Reported on page number: Page 12

If there were any deviations from the study protocol after approval was obtained please provide details of these changes in the Methods section of your manuscript.
Did this study involve local collaborators that are residents of the country where the research was conducted or members of the community studied? If you do not have any authors from said communities, please provide an explanation for this below.

Reported on page number: N/A

Except for one author (PBN) who is from India, the rest of the authors are from Uganda. During data collection, local and national traditional healers association (NACOTHA) leaders were involved in data collection and validation.

Everyone listed as an author should meet PLOS’ criteria for authorship and all individuals who meet these criteria should be included in the author byline, rather than the acknowledgements. Authorship criteria is based on the International Committee of Medical Journal Editors (ICMJE) Uniform Requirements for Manuscripts Submitted to Biomedical Journals - for further information please see here: <https://journals.plos.org/plosone/s/authorship>.

**Human subjects research (e.g. health research, medical research, cross-cultural psychology)**

Did you obtain written informed consent from a representative of the local community or region before the research took place? How did you establish who speaks for the community? Details of written informed consent obtained from study participants should be reported separately in the Methods section of your

We received approval to conduct the study in Uganda from the Uganda National Council of Science and Technology (Ref: HS1602ES). Clearance to collect data was also obtained from NACOTHA (National Council of Traditional Healers Association; NACOTHA, <https://www.nacotha.com>) and local leaders. The leaders introduced us to some of their healers, who were then contacted, screened, and interviewed.

How did members of the local community provide input on the aims of the research investigation, its methodology, and its anticipated outcome(s)?

The Traditional Healers, who are members of the local community, provided much input in the outcomes of the study by accepting to share their views about the care they provide, indirectly shaping the subsequent research questions and Validating the study findings.

When engaging with the local community, how did you ensure that the informed consent documents and other materials could be understood by local stakeholders?

The study tools and consent forms were translated into the local language (Luganda) that is commonly spoken in Uganda, which were approved by the ethical approval commitees. Interviews were conducted in the local language. Healers were free to ask questions about the study protocol before, during, and after interviews. Participants were also encouraged to withdraw from the study if they felt uncomfortable with some questions.

Will the findings of the research be made available in an understandable format to stakeholders in the community where the study was conducted (e.g. via a presentation, summary report, copies of publications, etc.)? Please provide details of how this will be achieved.

The Link to the publication will be shared with various stakeholders (E.g NACOTHA leaders) in the community where the study was conducted.

**Non-human subjects research using specimens/ animals collected as part of the study, or those housed in archival collections. Examples include archaeology, paleontology, botany and zoology.**

Did the permission you obtained from a local authority to perform the study include an agreement on access to outputs and benefit sharing? This may include procedures to enable fair distribution of the benefits and resources arising from the research performed. Please include any details of Prior Informed Consent and Benefit Sharing Agreements obtained. These may be required by field-specific regulations, for example the Convention on Biological Diversity (CBD) and the associated Nagoya Protocol.

Participants were reassured that all collected data or any plant specimen used for cancer management obtained were solely for academic purposes and that the research team was not using them for any financial gain. The following statement was inserted in the background section of the consent form, “*This study seeks information regarding your understanding of cancer as an illness and how you care for patients with cancer. This information will inform the development of policies, programs and projects geared towards promoting, professionalizing, and developing traditional medicine in Uganda*.”

Under the benefits section of the consent form the following statement was inserted

“This study has an indirect benefit you as a participant. The researcher hopes that the findings of this study may help contribute to the professionalization of traditional medicine through certification schemes and where possible may benefit from health education campaigns that may come thereafter.”

If the material used in your study was imported, please A) provide the year it was imported and B) indicate whether permits were obtained to import/export the materials used, C) provide details of any permits obtained. If this information is not available, please indicate this.

N/A

If you used archival specimens, please state how the material used in your study was acquired by the institute it is held in and provide details of any permits obtained for the original excavations/ sample collection. If this information is not available, please indicate this.

N/A

How was the potential cultural significance of the materials collected in your study to local communities considered in your research design? Were Indigenous peoples and/or local researchers and institutions involved with archaeological excavations / collection of specimens? If so, please provide a description of their involvement.

N/A

If your manuscript includes photographs of human remains please indicate whether authors obtained permission from descendants or affiliated cultural communities to do so.

N/A
