# Supplementary material for: The meaning of caring for patients with cancer among traditional medicine practitioners in Uganda: A grounded theory approach
Source: PLOS Glob Public Health. 2023 Jul 17;3(7):e0001764. doi: 10.1371/journal.pgph.0001764 (PMC10351711; doi:10.1371/journal.pgph.0001764)
Supplement: S1 Table — (DOCX) [file pgph.0001764.s002.docx]

| **Significant supporting transcript of quote** | **Codes** | **Major category** | **Memos/notes** |
| --- | --- | --- | --- |
| …the life of an individual is the most important thing, for money even if you get a lot of money and the drum gets filled up. You will never talk with that money; the money will never respect you or honor you. Money Is a visitor, money is a curse. Yes, we need it and it helps us, but money is not greater than an individual...It is not good to have your friend suffering(patient) because of money and yet you know the plants that can help that person. As God also helps. God willing that person will heal. Instead of leaving that person to die. For sure people, I help them for free. **[Tereza]**  I always put in my money and treat you until when you cure. After curing you will bring another person. That is where you benefit. Above all the unpaid balance is carried forward to another patient. If for example, I ask a patient 500,000/= shillings and they fail to bring all of it but pay 400,000/=. When they cure, they will bring another person, whom I will bill 700,000/=, but instead, he/she will pay 600,000/=. So, the other 100,000/= balance that the previous patient failed to pay has been recovered **[Rose]**  The patients pay slowly by slowly or bits by bits… He / She may give you 1 million Ugandan shilling for the start. He or she may start taking medicine (HM) and you may see them after 2 months, and they pay another installment, like that. You see cancer doesn’t affect those that are well off only, but also those that are not well off financially. **[Stefano]**  I tell you people keep laughing at me. I only charged him little money for going to pick up the medicine from the bush. If somebody tells me that I don't have money here, will you let that person die?  If he tells you that I am going to look for money! Will you leave him without medicine? The one who has money gives it to you (pays you), and the one who doesn't have money will say they are going to search for it. If he gets lost or never re-appears, let him go (no return) if he/she remembers you, he/she will give it to you **[Tereza]** | Advising the patient to pay in phases  Allowing the patients to Pay in phases or installments  Cancer contracting the financially disadvantaged (The TMP understands the patients they are treating)  Carrying balance forward to another patient  Charging little money to collect medicine  Combining treatment based on experience and ancestral instructions  Failing to pay for TMP’s services despite care after healing ((in all situations of paying little, promising & not paying, they are still happy with their roles because of the humanistic values instilled in them)  Giving an account of one’s actions to God/believing that everyone will account to God for their actions  Herbal medicine cures slowly  Keeping a long-lasting relationship (when the patient does not pay)  Marketing/Promoting the TMP  Prioritizing Life above money  Paying less for treatment  Receiving a token of appreciation  Providing free services  Returning with other illness & paying  Taking good news  Treating a God-given gift/ gift / not paying to learn  Treating all patients regardless of their financial status  Treating and paying later  Treating case by basis (individual basis)  Treating cheaply  Treating for free (not money minded/altruistic values)  Treating for free and hoping for customers  Treating for free and paying later (all-inclusive care)  Treating for free to attract more customers  Treating patients at a cheaper cost (compared to conventional care)  Treating patients for Free during the research (trial and error)  Treating poor patient  Unchanging dosage  Understanding the patient’s financial challenges  Helping the patient (providing free treatment-) | Prioritizing life over money | These are rational decisions the TMP Makes on daily basis depending on the type of patient they get per day. Particularly those that are poor (this therefore a rational decision on how to handle economically disadvantaged patients. So, the TMP prioritizes the patient’s life over money & the patient pays later directly or indirectly.  So many elements exist in this category, completely free care, partially free, paying in phases  The consequences of providing prioritizing life over money in a modern era were at times despised by relatives |
| When you manage a cancer patient, you should be ready to help people, but not feel bad about them nor look down on them or show feelings of disgust. It is like a calling you must be ready to serve the patient… **[Faridah].**  For me, I just accept that person because if you accepted "a calling to be a healer", you just must know that you will meet the such and such class of people. So, you manage each person in each category, in their way **[Nehemiah]**  You must have a strong/big heart because you sometimes see a certain wound and you start even fearing it. You must also believe in what you can do. You need to tell God to only make it easier for you. **[Faridah].**  The patients come towards you and explain all her problems and she/he has trusted you and then she tells you the in and out of their problems. However, they feel heartbroken when they hear that elsewhere, that person will say that all herbalists/doctors are bad even when others like us do not break confidentiality. **[Faridah].**  You may charge them expensively and they fail, and they end up dying, yet God gave you the gift and technology of using plants to treat his people, so God will accuse you of a crime you have committed. Yet did not even put in school fees to study it. Another reason is that I have some Godliness in me, I grew up without my real parents (grew up with a stepmother) if God did not help me survive. Would I have been alive? **[Jovia]** | A Calling to serve  Accepting any patient  Accepting Treating as a calling  Avoiding God’s accusations  their role as a “calling to serve”  Believing in yourself/ having confidence in your abilities  Calling upon God  Giving an account of one’s actions to God/believing that everyone will account to God for their actions  Having a strong heart while treating Fungating tumors  Keeping patient’s information confidential/Believing in keeping confidentiality as a golden rule  Living to serve  Not feeling disgusted when caring for foul-smelling sores  Our role is a “calling to serve”  Being Tolerant  Tolerating the foul smell from sores  Willing to help | Calling to serve humanity | *Developing the treatment philosophy*  These are intrinsic values or attitudes associated with treating or you need to help the patient to able to treat successfully. With these intrinsic values, the TMP can handle whatsoever challenges they face during the care of their patients.  Generosity |
| When he/she explains to me their illness, l makes them my friends. If he /she is older than me, I accept him /her to become my parents. Because how I would treat my parents is how I treat him/her. **[Sarafinah]**  There are also others (TMPs) who feel empathetic to patients if they have a religious heart. Then you ask yourself; “what if it is me who has gone and paid some money, and they do to me such and such things? What if he is my child or mother? that I have taken to the clinic, so if you ask yourself such and such questions, …you will find yourself not doing that.  So, you ask yourself if such and such a person was my relative, would I not have treated them? Or give them such treatment? **[Nehemiah]**  Any person even if they are not adherent but you talk to them about the illness and turn them into your friend…. they always change and take the medicine **[Nehemiah]** | Accepting any patient  Accepting/ accommodating the patient  Asking if it was my relative /comparing the patient to my relative  Basing on humanistic values to make decisions about patients  Basing on the feelings/humane values to make decisions about patients  Befriending the patient  Being courteous to the patient  Empathizing with the patient’s health situation / feeling for the patient / being empathetic  Feeling compassionate towards patients / treating patients with compassion  Owning the patient /Caretaking  Putting myself into the patient’s situation/empathizing with the patient  Putting self in patients’ shoes/ being empathetic | Embracing traditional social values in patient care | *These are culturally ingrained care values obtained socially from the community that the TMP instinctively applies to the care of the patients (collectivist social norms)*  *Embracing the values helps one to settle in their roles comfortably as seen in TMPs who espoused these values. They looked happy* |
| Second, you need to have gloves because the patient will come with a wound (sore). When somebody has a wound, it is clean? He/she must keep it clean; you cannot touch a patient's wound as a traditional medical practitioner when your hands are on dirty work or without gloves. So, you need to use gloves, together and maintain good hygiene **[Faridah].**  I make sure I have gloves, and I make sure that I, the traditional medicine practitioner, am clean, even when you are clean, the patients always feel at home (feel comfortable) or take their medicine comfortably with one heart or with a positive feeling or at peace with you **[Sarafinah].**  As you treat cancer, you treat it with fear of contracting it. If you have wounds, you are not supposed to treat (treat the patient with cancer), you can contract cancer why? Because the microorganism from a cancer tumor can also enter your sores. So, you end up getting cancer  When you are in your menstrual period you don't need to treat a person with cancer. You may be treating such a patient and when the time reaches you may go to clean your private parts and you may contract cancer. That is why they say that for a lady after treating a patient with cancer and you touch your knickers wash them, put them in the sun, and then wear them again after 24 hours. This even includes the clothes (uniform) you are wearing at the time of treatment. On the day of treatment wash them from the house and hang them in sun before using them again. Because the power of the microorganism that brings cancer would have weakened. **[Rose]**  I dry it outside but not in the ground, to avoid mud from entering it. Even something you eat or take; is not good to put in the ground. I use a tumpline and the reason is that I may say let me just dry the medicine on the ground, after all, it is not me who is going to take it, so I ask myself if it was me won't, I have gotten sick? Would I have taken it? Therefore, why do I give it to others? hygiene is the most important thing. **[Faridah]** | Applying herbs/medicinal plants after cleaning  Avoiding treating a cancerous tumor during the menstrual period  Cancer treatment taboos  Cleaning with gloves  Cleansing the wounds  Communicating illness  Creating a fresh wound  Differing in infection prevention and control (because cancer is contagious, so you need to take strict measures)  Drying clothes used during treatment  Drying medicine in a clean place  Ensuring asepsis during wound care  Facing the risk of contracting cancer/ perceiving cancer to be a communicating illness  Feeling comfortable/the patient's comfort (outcome  Getting infected  Gloving/ controlling cross infections  Indirectly advising/counseling the patient on poor hygiene  Maintain hygiene during wound care  Maintaining proper hygiene during wound care  Medicines are used concurrently with proper hygiene /Linking hygiene to medicine effectiveness  Pouring hot porridge on the wound  Prioritizing hygiene during drying  Receiving patients with poor hygiene  Throwing away gloves/removing gloves after wound care  Traditional ways of asepsis (changing practice-no long in use)  Treating while fearing contracting cancer  Understanding the importance of ensuring proper hygiene/ the patient’s comfort | Prioritizing hygiene during patient care | Wound care principles and process of care  Managing a dirty patient with humiliation (Not/avoiding humiliating the patient)  Basing on cultural principles |
| I used to see such people with cancer for a long period but by then I was not much involved in the treatment.  I used to see my grandparents giving certain treatment in the village and I used to ask my grandparents to tell me, why she was giving that medicine to such people. I would also ask her, that one with such a wound or sore what happened to them? He /she has swollen legs. She would tell me it was cancer. I used to ask her (my grandparent) what type of medicine do you use. She used to say that am still young, but once I grow up or become old enough, she would then tell me that this does this and the other that. If you use this medicine, this is what happens. **[Yozefinah].**  So, our grandparents were so keen on us learning the medicine and so he/she would say go and pick such and such medicine we boil it and give to that person and would heal (of cancer) **[Hajji Hassani]**  I don’t know but I used to hear my grandparents say that throat cancer is so bad always be careful about it,  If you get a person who has cancer of the throat, start that medicine early but also she used to say that such and such person needs to first go to the orthodox doctors. She used to say that somebody may chew certain herbs and if it is tonsillitis it will disappear, but if the pain does go away then it is cancer of the throat cancer.  My grandmother did not give me the details of the diseases. Long ago people would only tell you the medicine and what it treats only. Instead, they would tell you, do you see plant this and this? It can cure this and this disease. You prepare it like this and this. If a person comes with such and such symptoms, you give it to them. For him/her, he would have finished teaching. And that is enough. But they would not go into details to explain to you. Only disease, the plant it treats, and how to prepare it. Were the only things told to us. Except, my grandparent told me while preparing/ boiling that medicine, it is not good to allow it to pour and its cream should not pour down. You should keep sitting around the fire until it is well cooked. You keep steering it like you are preparing ground nuts soup so that the foam or cream doesn't pour down. She also told me that the stem back is very important to cook with it as it has a lot of value  **[Tereza]**  None of those groups (traditional healers' group) helps us in cancer care. By the time you start treating pts with herbal medicine, you must have expertise in herbal medicine **[Silvia]**  But when I contracted it attacked the whole breast and only fluid came out of it (not pus) and milk, when I went to the hospital by then my grandparent had not come to visit me and they told me that they were going to take me to Mulago hospital and chop off my breast. When she had that she said to people at home to put her on the bicycle so that she could come to see her daughter, when she came, she told me not to go to Mulago Hospital to cut off my breast. I am going to treat you and you will heal. I am going to give you herbal medicines and you will heal. The disease you are sick of, I understand it very well, don't go and they cut off your breast so that you have one breast, she then brought me herbal medicine at the hospital and instructed me to use it, at the hospital take it (drink it) and other apply it directly on the breast. Within one week the breasts stopped leaking, and within the second week the milk stopped leaking, that's when I learned that cancer can heal. I also learned medicine and I now use it. **[Tereza]**  For example, I got sick for 9 years from 1997 to 2006 when /I was sick, I moved to all modern hospitals, and at the start of my health care seeking I was without illness but by the time I had reached 7 years, I had already contracted cancer of the intestines (colon) Like me I was sick of cancer, and I confirmed that I am going to die and I was very sick. So all people I was moving with for treatment all died. I had cancer of the intestines, some had liver cancer others kidney cancer, and all those died. The time came and I understood that life was in food, in eating and drinking the right food and drinking. I started taking them but there is no place I didn't go to, I started in Kiboga then Mulago, and after I went to Rwanda, they used to refer me to orthodox doctors, but cancer failed. I then confirmed that my life is going (I am going to die) thereafter, I started to make research to find out why people get sick and die. When I realized that food played a great role, I started changing my diet and now today I am cured, and I am treating other patients. [David]. | **1. Learning from ancestor/predecessor:**  Ancestors characterize cancer as a swollen limb  Basing on the Duration and speed of spread and odor  Describing the Medicine harvesting practices as instructed by the ancestor  Emphasizing proper cooking /preparation of medicine  Instructing in treatment, preparation, and symptoms, not disease causes (reason for little knowledge)  Instructing TMP in the investigation (before treatment)  Instructing/ teaching about severe tumors/cancer or disease severity  Involving children in patient care/ learning through being involved)  Observing ancestors diagnose cancer (Traditionally investigating cancer  Observing for a chronic sore on the leg  Obtaining plant knowledge from predecessors /learning from predecessors  The predecessor sent us to pick medicine  Receiving instruction and treatment from predecessors (learning through experiencing cancer-tell this story)  Receiving instruction from predecessors (about types of cancer and how to prepare medicine)  Receiving teaching from parent  Seeing ancestor smearing it on the wound  Seeking information about disease symptoms (asking)  Seeking information from predecessors (limiting information to age)  Seeking information from predecessors /Asking to know the diagnosis from Predecessors  Timing of harvest  Traditionally investigating cancer  Traditionally treating cancer  **3. Learning through ancestors treating the TMP with cancer**  Failing to improve on conventional medicine  Referring for surgery  Predecessor intervening and Giving herbal medicine  My ancestor ordered me to apply medicine to the breast sore  Improving symptoms  The predecessor ordering the patient to take herbal medicine alongside chemotherapy  Predecessor Preparing for me concoctions for drinking, and bathing  Predecessor Ordering me to Mix herbs with oil and Applying them on the skin  Receiving instruction from my ancestor  Seeing ancestor treating  Predecessor Healing me (the concept of healing originates from their observations of their ancestor)  **3. Learning through the cancer disease experience/TMP self-treating**  Experiencing imminent death  Understanding the role of food in cancer  Making research  Healing  Treating patients/ using own experience to treat cancer  Starting to treat with basic expertise | Initiating the TMP into the  predecessors’/  predecessors’ practices | *Developing the role of a healer*-pre-practice  These are ways TMPs learned how to treat cancer in pre-practice years, usually under the guidance of their predecessors. The predecessors mainly provided instruction in fewer areas including medicine, preparation, and symptoms.  During pre-practice years, during the early years of life in terms of the age  This denotes traditional beliefs about the cancer diagnosis. Here the TMP explains how their ancestors diagnosed cancer, or their traditional perception of cancer especially picked from predecessors. These partly influenced how TMPs diagnose cancer today or observe for signs and symptoms of cancer |
| It started with my grandfather, let me say my father was a traditional doctor (TMP) and he died. There are things before he died, he used to show me a few of them and after he died, I inherited his role **[Hajji Hassan].**  Our medicine is ours; it is inherited from our grandparents or inborn medicine **[Matia].**  One time I was sleeping and my ancestor through dreams asked me if I would be a herbalist, I told them yes then they started showing me plants in dreams and also taking me around and showing me the medicine **[Solomon]** | Appointed to treat by predecessors (chosen by predecessors )  Inheriting my parent’s role  Inheriting the role of my father  Performing our grandparent’s business | Inheriting my predecessor’s role | The appointment and inheriting the role of the TMP seem to be the bridge between pre-practice to practice years for those who learned through their predecessors. |
| I am always on the radio and TV teaching people the role of food in cancer. Therefore, God hid life in food, all human life is in food (natural food). after hiding life in natural food, whoever gets that food has life and whoever misses that food he /she is sickly **[David]**  I tell him or she lets go to the hospital, I can even escort him or her so that we can go for counseling together….He or she becomes part of me; because lam the one who has been his or her doctor, so I escort him or her to the orthodox healthcare workers, they ask what happened, and I then explain to them that he/she has used herbal medicine, but I have failed to treat them… ..because even when went to Hajjati Janati (a herbalist-not real names) she said the cancer was not the problem.…… I then told her to let go to Entebbe hospital and she started her treatment again **[Faridah].** | Communicating to masses (health educator-new roles)  Escorting patients to the hospital (Dual roles of TMP & caretaker)  Escorting patients to another TMP  Escorting patients to the hospital  Escorting the patient to another herbalist  Escorting the patient to the hospital to confirm a cancer diagnosis  Explaining the patient’s treatment history & condition to orthodox doctors (transitioning care, as caretaker) from TMP to orthodox | Evolving TMP roles | Once they started practicing their roles, they have been expanding from dispensers to mass communicators thanks to other enablers like telecommunication, at times they act as caretakers. |
| I harvest herbal medicine and manage cancer like I was taught by my grandparents **[Hajji Hassani]**  When I go to the field (wilderness or bus to harvest medicine) I go with coffee, and I serve the wilderness because it has its owners. Or I put 100 shillings on the and harvest the medicine so that I have bought the medicine to help and heal my people. **[Faridah].**  After getting the plants from the villages and after gathering all the plants that I think can heal one with cancer, I bring them and pound them separately and I cook each plant alone (separately) and then mix them after cooking them…to do that, I also based on what my grandparents used to do **[Jonan].**  My grandparent told me that if a person has a growth in the urethra, even then, you get that medicine concoction that keeps dropping in the urethra. You see the person getting cured. He also keeps dropping in the urethra while also taking it orally. So that is what I do **[Tereza]**  I can't admit them all I give them medicine because I can't manage it financially. That I bought a hospital bed and I build a hospital/clinic and I am now giving medicine, so I give you and you go to your home so go and treat yourself from that side. So, treat yourself alone, I don't have admission beds.so I don't have where to put them because I don't have money to build for the patients where will stay, but if I get the financial ability I can get where to put them. **[Solomon]**  First and foremost we lack money to register the medicine. National drug authority usually charges between two hundred thirty thousand to four hundred thousand to approve my medicine after approval it’s up to you to work or not **[Silvia]** | Basing dosage of medicine on ancestor’s experience  Basing on ancestral directions  Grinding, boiling, grinding, and selling HM  Harvesting like our grandparents  Mixing/dosing according to ancestor’s experience  Paying /serving the wilderness owners/ predecessors / Spiritual rituals  Similar medicinal picking practices  Providing similar care  Sun Drying  Wet Drying  Lack of finance for building a clinic  Lacking funds  Low profitability  Not advertising self  Rich patients visit popular TMPs  Patients may not have the finance to pay \  Lacking funds to register medicines  Preserving with natural preservatives  Lacking machines to process medicines | Maintaining their predecessors’ practices | Is it practicing like ancestors or basing on their knowledge to innovate or both depending on the circumstances? Practicing like our predecessors |
| The other difference is that a person with cancer has his or her own unique medicine. I make sure that the medicine for cancers is available before they come that is why I always want cancer patients to call me before they come to see me so that I get to know if I have the medicine or not if it's not available, I make sure I prepare it early in time, so I always want to make a special appointment for them and if there is medicine, I re-schedule the day and I tell them to come on such as such a day because for such as patient, I don't want them to find difficulty in that illness **[Faridah]**  There has been no change in care during this COVID-19 pandemic. We have been collaborating with our patients (leaving our medicine for pts to pick). We have our delivery point where we dispense our medicine. In addition, we used motorcycles to deliver the medicines **[Silvia].**  So, for me, I do not have any taboos, and whomever I give my medicine it has no taboos  We shall also show you the combinations of the medicine if you give us money **[Hajji Hassani]** | Behaving in a dishonest manner/ Valueless TMP  Collaborating with patients/leaving medicine at central points  Making treatment combinations Colon cleanser herbal medicine (first line), then HM for cancer  Delivering medicine to the patient  Differing in customer care  Differing in diagnosing cancer and comorbidities  Differing in the frequency of communication  Differing in medicine taken  Differing in packing herbal medicine  Dishonest TMPs (adaption to market forces)  Innovating in adversity -adaptation  Making prior appointments / Preparing medicine before a visit  Not delegating cancer patients/ Opening up a few branches  Prioritizing the patient over taboos  Providing treatment regardless of the covid19 restrictions  Sending medicine to patients/Avoiding Medicine going bad  Simplifying the patient’s life  Treating with fake products.  Using approved medicines (Adapting to changing environment during practice years)  varying instructions/ my Ancestor’s direction  Varying medicinal picking practices | Evolving practices | Differences in care across TMPs-indicates modernization-There are technically those that are moving out of the business, those consolidating or those settled in their roles, and those adapting to the changing environment- along winding road  Uniqueness in care across TMPs  Uniqueness in care across conditions  Providing urgent and unique care  Innovating during adversity (COVID-19)  Developing combinations and packing medicine  Differing care across the TMPs  Differentiating the care of patients with cancer from other conditions |
| Like you have come to do research, likewise, I also sit down, and I say this and this plant based on previous use, by grandparents, if combined with this, it can cure this and this disease and if it works out, I give it to somebody else. **[Jonan]**  If a person comes with certain cancer but also with symptoms of HIV or any other illness and I am not sure of the right treatment, I mix in herbs for all illnesses I suspect (like HIV, anemia) and once it works, I use the same formula to another patient who presents similarly. **[James, 40-year-old herbalist, central Uganda]**  We use this knowledge of those men (orthodox doctors or western doctors), for them they understood, that the things we must use are the plants. When we used them, they worked, and we understood them. But these books are so expensive, these books where I get the knowledge, they are expensive (open a book on nutrition and herbs and show me the plants She uses to treat cancer **[Annah]**  Even when they are using herbal medicine, if I am near an orthodox health care worker, I don't hide away from him or her, I turn him /her into my friend, because there may be counseling, he or she may add to me and in the end, I add it to my patients. It would have been good that a herbalist and an orthodox doctor to work together because herbal medicine is where the modern medicine (tablets) we use comes from **[Faridah]**  Yes, I was taught by my parents, grandparents, and my mother (who still today practices and treats using herbal medicine) in fort portal me. I don't have any spirit on me but when I sleep I may dream of a certain tree and instruction on how to use it for treatment of cancer and I go and do it and it works for that person **[Jovia]**  But when we went there together, after more tests, we found out that she had many other illnesses and they told me, maybe because of all those other illnesses, is the reason why she failed to improve so she gets treatment now at Another herbalist's treatment site. **[Faridah]** | Assessing healing during research  Assessing the dosage during research  Assessing the period of effectiveness during research  Basing dosage of medicine on outcomes of previous patients  Basing on the previous treatment experience  Befriending orthodox doctors and learning from them  Combining treatment based on experience and ancestral instructions  Conducting more research  Dreaming medicine/ Obtaining new medicinal knowledge through dreams from the ancestor  Enhancing knowledge through reading  Expensive books  Finding out if herbal medicine works  Incurring the cost of research  Learning how to counsel the patient with cancer from orthodox doctors  Learning more about treatment failure from other TMPs  Mixing according to results of own previous experiences  Mixing medicine for various illnesses  Treating a patient by trial and error  Treating using experience (learning how to treat from experience/)/Trial and error  Treating while conducting research  Trying the medicine  Understanding the dangers of concurrent use of HM and chemotherapy | Continuously acquiring new knowledge based on circumstances | Still, during practice years, their knowledge of medicine kept on expanding through communication with predecessors via dreams, trying out trial and error, and using that experience (properties). Given that the concept of cancer & disease manifestations kept changing TMP had to adapt to the situation by learning how to treat various cancer. This required them either to consult their predecessors or try out various treatment options. Occurs during practice |
| In most cases, once I receive sick patients who claim to have cancer, my role is to find out, how and when they got know that they have cancer. Afterward, I ask them to provide me with the medical reports for my perusal, either from home or at the hospital. **(Faridah, 38-year-old herbalist, central Uganda)**  The way I started treating her is that she came and explained to me how she was feeling bad (her illness symptoms), after explaining to me all symptoms, I told her maybe, she may have contracted cancer, but then she told me, that she didn't know that! I then told her to check her breast (to do a self-breast examination) please first, she then told me that she does not know how to do a breast self-examination. I told her to lay on her back and start pressing her whole breast softly, I told her to feel for any stone-like mass, that is movable as it is an indication of breast cancer. So, after she identified that stony-like mass, I told her to go to the orthodox hospital for further investigations of that mass. I do this all the time I get a patient with such breast symptoms. Because that stony-like mass is not a good sign  Okay for the patient with cancer of the breast, I may touch the breast and examine it if I am the first contact and then I get to know that probably it is cancer if I am the first to see the patient.  But even if I have felt that it is cancer, I always feel my examination is always not conclusive, and that is why I first refer them to doctors.  Therefore, it would not be good to start any person on any herbal medicine or any other treatment without their medical records that show that such a person has cancer. Why? It is because of what I told you earlier that I have no machine to check you and there many diseases that are similar in presentation signs and symptoms that appear like signs and symptoms of cancer. So I need to first prove that you have real cancer before I can start you on my treatment after I have understood what I am treating. But because I may not be understanding what I am treating, herbal may become difficult to heal you. I don't know how long cancer you have has taken (I don't know if you have been diagnosed with cancer) because there is a treatment that I will give you when your cancer is "late stage" or "old or mukulu”  **[Faridah]**  In the beginning, if my patient continues to use my medicine (swallow bile duct or chew certain plants), if the person has cancer, signs will appear (like yellow eyes or throat pain). **(Rose, 40-year-old herbalist, western Uganda)**  So, I can know one who has a spirit and one who doesn't have it. For example, someone may come and say that he is posed by a spirit Kiwanuka (fire), and yet I as a traditional healer am also possessed by the same spirit. So, I know one who is possessed by the same spirit, how they behave for example I can talk to that spirit about what it wants, for example, it asks alcohol. I will know that is not the spirit because Kiwanuka doesn't ask for alcohol or smoke. I will also ask the spirit where it comes from. And if it says Singo, that would be wrong since I know where Kiwanuka comes from **[Sarafinah]** | **1. Obtaining history and medical records**  Asking for medical records  Asking to explain symptoms  Asking when and how of their claims (taking/obtaining brief history)  Basing on the hospital's investigation  Basing on medical records to prove cancer diagnosis  Basing on medical records to diagnose and classify cancer  Basing on orthodox doctors for diagnosis  basing treatment on orthodox doctors’ diagnosis  Brief history taking  Claiming to have cancer  Coming/presenting with medical reports  Coming/reporting to TMP with medical forms  Explaining the disease symptoms  Fast developing wound  Interpreting medical records Using a third party -may affect privacy  Knowing the cancer type before the treatment/treatment according to the cause  Obtaining brief health history/asking patient to narrate disease history/narrating disease histories  Receiving already investigated patients  Receiving patient Claiming to have cancer  Referral to orthodox doctors for investigations or cancer diagnosis  Reporting cancer diagnosis  Telling/ confirming cancer  Treating all healing cancers after investigation irrespective of pt characteristics  treating with a clear diagnosis  Understanding the type of cancer after investigations by orthodox doctors.  Using experience to conclude on cancer type  **2. Observing the signs of cancer**  Basing on observed signs  Differentiating cancer symptoms from other illnesses  Differentiating cancerous sores from other sores  Observing the signs and symptoms of cancer  Observing the signs/symptoms of cancer/Suspecting cancer (Observing the clinical features of cancer)  Observing their skin  obtaining the brief health history  Suspected non-healing tumor  Suspecting cancer  **3. Examining the patients**  Directing self-breast examination  Examining some patients with cancer / of same-sex /fear of examining opposite sex (during same sex)  Examining the patient  Examining the patient (characterize cancer after examination)  Fearing rape  Suggesting self-examination of the breast  **4. Confirming cancer using plants**  Confirming cancer by treating it using plants or animal products  Confirming cancer with animal products  **5. Diagnosing spiritual problems**  Increasing cancer frequency and type  Preventing cancer/settling through treating it  Ruling out another related illness/avoiding overdose/side effects  Starting them on treatment after confirming the diagnosis  Treating a clear condition (consequences of confirming cancer | Confirming the type of cancer disease | Describes the process or steps the TMPs used to confirm that the patient had cancer before they could provide treatment.  The outcome of this is that they can start the patient's *treatment with a clear mind in their heads* (treating with a clear mind or conscience)  This seems to be the bridge between confirming and treatment of cancer (are they treatment principles) |
| They brought her when she was in a very bad condition with cervical cancer when she could not move or walk when she cannot do anything when she could only sleep. She could sleep all the time. When she could sleep, she could not want to put her legs together. She could sleep when her legs are standing (rhizotomy -position) or she was fowl Smelling **[Tereza]**  I have lost one patient, but I don't know whether it was cancer or not, because she was old (over 87 years) and had intestinal cancer. She had spent a lot of years under treatment, she came when the people of Mulago hospital had tried their luck several times and failed, so she came here as a last resort **[Karoli]**  Yes, of course, we treat poor people, rich people can't come here to obtain medicine from us [Jonan]  They took him to the hospital here and abroad, but they failed to treat and heal them after some time they came back to me but I refused to start the treatment **[Hajji Hassani]**  A person comes to me knowing that they have cancer, but they don't believe it is a physical illness except they believe it is caused by family spirits. Some come saying that in addition to their cancer, there is also witchcraft that is involved in their illnesses **[Nehemiah]**  There are few patients from here but most patients I treat I get from those whom I have healed/cured **[Stefano]**  Most patients came from far **[Silvia]**  No, I have never got patients that are very sick. I always get patients that can talk, cancer can catch a patient that is normal and such a patient may come to me for treatment or they bring the patient when they are worse off. If they bring a patient when they are worse off I cannot even treat him or her because you find that they have not checked him or her (investigated for cancer [Hajji Hassani] | Avoiding severe cases  Cannot swallow  Characterizing patients that visit the TMP /patient attributes  Characterizing patients with advanced cancer according to the treatment procedure  Characterizing cancer according to symptoms (describing the characteristics of the various cancers)  Characterizing cancer according to type  Characterizing patients with advanced cancer according to age >50yrs., cancer has spread throughout the whole  Characterizing patients with advanced cancer patients- old, discharged  Classifying according to disease severity  Comparing Advancing visav nonadvanced  Discharged patients from the hospital  Exhausting all other options/patients visiting TMP's clinic as the last option  Failing to heal  Likely to be amputated  nonsevere cases  patients Not walking /sitting  Obtaining discharged patients  Passing foul-smelling discharges  Previous patient referring/ Calling TMP  Receiving current patients referred by previous patients (thru Patient sharing telephone numbers)  Receiving financially disadvantaged patients  Receiving hospital treatment failures  Receiving patients in stable conditions  Receiving patient referrals /treatment failures in hospitals  Receiving patient referrals from orthodox doctors  Receiving patients with advanced cancer  Receiving patients in worsened conditions.  Receiving patients seeking assistance  Receiving patients with spiritual perceptions or perceptions that their disease is due to spirits  Receiving a referral from previous patients (patient-to-patient referrals)  Receiving referrals from Orthodox doctors (in)  Receiving self-referred treatment failures from hospital/ discharged patients  Receiving treatment as a last resort  Receiving worse and more hopeless patients (advanced-stage cancer)  Seeking cancer treatment from TMP following medical diagnosis (self-referral)  Sick for quite a long time  Sickly/bedridden  Sleeping, Bedridden  Sourcing or searching for patients with cancer  Telling the patient their prognosis before treatment (advanced preparing pt expectations)  Treating as the last hope/option  Treating distant patient  Treating economically disadvantaged patients or poor patients  Rejecting a patient with cancer esp of the private parts  Receiving patients with reports of treatment ineffectiveness  Rejecting some patients | Characterizing and categorizing patients with cancer | Characterizing patients received, modes of receiving patients for care  Forms of referral  (You may need to separate referral into two: in and outside referral)  Referral from orthodox doctors is one example of referrals  Receiving patients with cancer |
| I always pick, boil the medicine and pack it for them. If we suspect that the medicine might go bad, we put in natural preservatives from other plants or animal species or African preservatives **[ Hajji Hassani]**  In most cases, cancer patients are stigmatized or rejected, and people feel like those patients should not come nearer to them (feeling disgusted by patients with cancer) like patients with cancer of the private parts. Since it presents with a foul smell, they reject that patient and whatsoever the patient with cancer touches nobody wants to touch it even if it is a cup. So they say if I take many types of medicine they will start belittling me by my illness (talking badly about me) whenever I am making my medicine, so I decide as a traditional medicine practitioner that let us mix it so that patients take one jerrycan of medicine instead of many **[Nehemiah]** | Absence of superstitions during medicine preparation  Adding natural preservatives in HM  Avoiding Medicine going bad (rationale for above)  Avoiding losing nutrient  Avoiding night harvests due to risks/hazards  Avoiding stigmatizing cancer patients  Basing on the number of plants to determine the dosage  Boiling  Boiling and drinking medicine  Boiling and packing medicine  Collecting and preparing HM  Collecting medicine  Cooking them together  Drying herbs  Drying medicine  The patients returned to pick up the medicine  Explaining medicine preparation practices  Exposing herbs to early morning sunshine.  Gathering plants  Grinding herbs  Giving the patient antioxidants  Harvesting any season  Harvesting plants  Harvesting plants on demand  Harvesting practices  Keeping medicine for emergency  Killing cancer-causing germs  Law of Signature language  Mixing multiple comorbidities treatment to avert stigma  Making powder  Mixing in water  Mixing later/ making a dose  Overdosing has S/E  Pounding and cooking separately  Preparing and Giving medicine to the patient  Preparing herbal medicine for the patient  Prescribing dose  Preserving in dried natural form  Process of preparing medicine  Requesting funds from patients to pick medicine  Shaking to ascertain the concentration  Sieving and putting in a jerrican  Storage practices  Storing process  Testing it before giving it to the patient to check toxicity and avoiding arrest  Refiling medicine continuously  TMPs decide the plants to mix  Understanding and mitigating negative community attitudes through combining treatment  Using and mixing multiple plants  Using powered HM (preparation practices) | Preparing medicine for the patient | During patient care (before & during subsequent visits), the process of care, |
| so, you manage each person in each category, differently, the poor and those who are well off **[Nehemiah]**  For patients with cancer in their private parts (cervical/uterine), I always give them medicine for drinking, as well as for the silt bath. **(Tereza, 84year-old traditional healer, midwestern Uganda)**  It depends, some patients have a belief that they don't like western medicine and that it has side effects, it makes them eat a lot yet sometimes they don't have money for food yet our African traditional medicine act as food supplements as well as medicine. That is the chance we have when a person uses it, and they don't get an urge to eat food anyhow. So, for such people, we give them herbal only. The body remains getting exactly what it needs to be treated and as well as it gets the necessary nutrients to remain in the body yet western medicine treats but does not add nutrients to the body or leave the body deficient of the nutrients. That means others take both herbal medicine and chemotherapy **[Kalori].**  I give medicine that's all. I give them one mug or cup (500mls) which they take in 3 divided doses. In the morning, middle of the day, and in the evening after they have taken food, If she/he doesn't eat, then they don't take the medicine. The other medicine they put in the milk, if they don't put it in the milk it actually may cause harm to them. If she/he doesn't have milk, then they don't take the medicine. **[Hajji Hassani]**  For some patients, I can give them some herbs only to reduce a particular side effect but not to treat cancer. So, I give them to reduce the vomiting, I also give them a little plant and I tell them to keep chewing it the vomiting will stop without killing the western medicine dosage **[Jonan]**  They go to the hospital so that they investigate (orthodox doctors) whether this cancer is from micro-organisms or for ulcers or it is from this and this and that is my basis for treating these patients **[Hajji Hassani]**  My line treatment is counseling, apart from counseling there is also medicine that I give you at the beginning, but as he moves on, the condition of the patient is the one that indicates or shows me either to add them herb or another like when you to go to the hospital **[Sarafinah]**  if somebody tells me that there are so many sores, I don't want to insist on staring them at treatment. If they are so many sores, even you see that it is worse. I start to ask myself if it's my daughter/son or parents, when he /she has such by sores, I say no go far with orthodox medicine or lets us use both orthodox medicine and herbal medicine. I then tell them to go to the hospital and they give them treatment (chemotherapy) as I also give them my treatment (herbal medicine)  Also, there is the treatment that I will give you when your cancer is still late stage "old or omukulu" but if I give you the first line treatment, your cancer is late stage. But if they screen you and tell you the size of your sores "cancer stage" then I can know which type of treatment to give you. **[Faridah]**  When I was treating him with all the treatment (herbal medicine and chemotherapy) the medicine grossly weakened him, he wanted to die so I told him to let's treat one by one. they started using herbal medicine only. so, the pt choose life and so we treated it with herbal and herbal healed it.  I also get to know that she also takes the other medicine (chemotherapy) at 10 pm, so I tell them to take mine at 4 pm. Why? Why do I give him /her that gap of 4 hours plus is because, I don't want my medicine to find the other medicine in the body and they crush each other (fight each other) or they interact with other?  **[Rose]**  The care for the two does not differ much because we said that if we find advanced cancer, there is a likelihood of dying from cancer despite the same treatment. but if the person was early-stage cancer the person is likely to cure. Because all are treated with food. What treatment treats "early and advanced stage cancer" is still food (nutrients) from God-created plants **[Silvia**]  The prostate cancee is treated for two months, the tumor one and half months, colon cancer takes about six months [Stefano] | Adding rehydrating plants  Adding to concoctions certain plants that chase away spirits  Advising the patient to either use chemotherapy or herbal medicine (vs alternative)  Advising the patient to take herbal medicine alongside conventional therapies/ medical pluralism  Advising patients to take medicine after eating food  Advising the patient to return for Refilling of medicine  Alternating between chemotherapy and herbal medicine (pts seem to die when taking his herbal & conventional)  Alternating herbal with conventional  Applying intravaginally  Applying medicine to the wound  Applying medicine topically  Applying the powder to the wound  Applying topically  Asking about eating before the medicine  Asking about the period between food and medicine  Asking about the quantity of intake  Avoiding raising blood sugars  Basing on medical records (stage of cancer) to treat cancer  Basing on an orthodox doctor's diagnosis or judgment to start on treatment or refer (when)  Basing on the patient's condition reflected in medical records to treat cancer  Basing on patient conditions to treat them  Basing on the patient's condition decisions to determine the dosage  Basing on the patient's condition to decide when to start treatment  Basing on the previous treatment experience  Basing on the stage of cancer to determine the treatment period  Basing on symptom severity to determine the dosage  Basing on the patient’s condition to increase or taper the treatment dose (number and type)  Basing on the patient’s condition to order the dose  Basing on the patient’s conditions to determine the drug dose.  Basing on the patient’s stage of cancer to start treatment  Basing on the type of tumor to determine the Treatment period  Basing the dosage on intake of conventional therapy  Basing the dosage on the patient’s condition  Basing treatment on medical records and stage of cancer  Basing treatment on the stage of cancer  Becoming poisonous  Believing western medicine is also from trees  Calling and sending medicine (unique/ no need for reassessment and lines at the hospital)  Caretaker/ ensuring treatment adherence  Caring process for those with early-stage cancer (not weak)  Changing and giving other types of HM/plants  Changing occurs according to the part affected  Changing treatment based on improving health  Cleaning the wound  Depending on improvement  Differing in the period of intake of HM  Differing in recommended dietary habits  Discouraging use of conventional medicine  dispensing HM  Dispensing medicine to patients with cancer  Distancing from cancer patients  Drinking and applying the medicine  Drinking medicine  Drinking milk  Drying up wounds  early visav late-stage  Early vs late  Exceeding recommended dose  Explaining mechanisms of HM  Feeling good when the patient follows orders  Finding no trouble in taking HM and conventional treatment (from experience)  Focusing on eliminating the cause of the symptoms  Frequently receiving patients returning to pick up treatment  Giving foods that replace water and blood cells / replacing body water and blood cells first  Giving medicine  Giving medicine to patients in distress without a diagnosis  Giving them medicine  Handling/treating according to patients’ presentation  Healing period  Herbal medicine acting as food supplements  Herbal medicine acting for a longer period  Herbal medicine adds nutrients to the body  Increasing plants  Increasing the dose  instructing alternating treatment and timing  Instructing the patient on the dosage  Instructing the patient on how to insert medicine intravaginally  Instructing the patient to apply HM topically (specifying advice to the type of cancer  Instructing the patients on dosage  Instructing to take plenty of water regardless of the type of cancer  Instruction on taking herbal medicine  Interacting with herbal medicine  Killing germs that cause cancer slowly  Life centers in the abdomen  Long-acting for longer times  Longer half-life  Medicine causing/re-awakening of dormant ulcers by traditional medicine  medicine that treats internal and external wounds (cancer)  Mixing herbal in jerries and smearing (specifying advice to the type of cancer)  Mixing more trees leads to concentrated medicine hence less dose  Mixing multiple plants in concoctions  Moving slowly (slow but incremental effects)  Never mixing herbal with conventional medicine  Not mixing herbal and western medicine  Ordering cancer patients to take animal products  Ordering patient to Put medicine in milk  Ordering patients to use herbal medicine alongside cancer conventional therapy  Ordering the patient to cease treatment after confirming the healing  Ordering the patient to use oral medicine and siltz bath  Passing medicine under private parts  Passing watery diarrhea  Perceiving cancer to heal through the consumption of sour foods  Perceiving herbal medicine as medicine for cancer and nutritional supplement  Perceiving herbal medicine to work gradually.  Picking packed medicine  Prescribing herbal medicine according to cancer type  Prescribing herbal medicine to patients between diagnosis (before diagnosis- an exception)/  Prescribing intake of herbal medicine and for siltz bath  Prescribing medicine  Prescribing powder medicine  Prescribing specific plants for the symptoms  Prescribing the medicine  Prescribing medicines for wounds  Prescribing/ ordering the patient to take plenty of HM to replace fluids  Prescribing medicines/killing cancer-causing germs  Prescribing/continuing with anti-cancer HM later  Prescribing/giving /treating with healthy foods first  Process of care  Providing treatment  Recommending conventional therapy over herbal medicine  Reducing the herbal medicine dosage when taking conventional treatment  Restarting on treatment  Scheduling for the patient when and how long to take the medicine  Scheduling to take herbal medicine alternating treatment it with conventional therapy  Self-reporting their conditions/brief history taking  Sending medicine to patients  Smearing on the cancerous skin  Some mixing herbals with conventional treatment  Stopping certain food  Stopping conventional treatment and using herbal  Supplementing the body with nutrients  Taking herbal and conventional treatments  Taking herbals first before chemotherapy  Taking herbal medicine for a long time  Taking herbal medicine in the late stages of chemotherapy  Taking HM for longer periods  Taking a long time on care  Taking medicines orally  Taking treatment orally  Treating and caretaking patients at the TMP's home  Treating the patients from TMPs homes (dispensing and patient cooks & self-administer, not very sickly)  Teaching the patient how to use the medicine  Teaching about medicine use  Telling patients about treatment phases (expectation mgt)  the process of handling adverse side effects  Treating /caretaking patients at their homes/ providing patient home care (those who could afford services)  Treating according to cancer type  Treating according to the cancer patient's symptoms  Treating after hospital investigations  Treating and believing in the healing GOD  Treating and healing precancerous states  Treating blindly/ without knowing other illness  Treating cancer or treatment symptoms  Treating cancer and spiritual problems separately/distinctly  Treating cancer for longer periods  Treating cancer using animal products-bile  Treating case by basis (individual basis)  Treating him for a short time following chemotherapy  Treating internal and external cancer  Treating patients with herbal medicine alone  Treating patients with herbal medicine during chemotherapy cycle  Treating symptoms  Treating advanced cancer and healing  Treating the patient when close to the clinic (advanced)  Treating the patient with little patient’s history  Treating them from their home (no close monitoring  Treating using nutritional therapy  Treating visible signs and symptoms  Treating with food  Treating with herbal medicine  Treating with little knowledge about the right dose.  Treating with no rules  Treating with the hope of the patient either improving or not/Explaining treatment probabilities  Treating with the same medicine regardless of the cancer stage  Treating without confirming or suspected cancer cases.  Treating without the knowledge of patients using conventional therapies  Treating with herbal medicine with little knowledge of the use of conventional therapies  Understanding the dangers of concurrent use of HM and chemotherapy  Unaware of duo intake of HM and conventional treatment  Using a single therapy to treat multiple illnesses because of similarity in presentation (presenting with wounds)  Using different products for each cancer  Using herbal medicine alongside surgery (medical pluralism)  Using herbal medicine gradually and continuously  Using medicine that heals wounds to treat cancer  Using the same plants to treat Multiple illnesses  Waiting for God’s mercy/  Washing under the private parts | Individualizing care | Included roles before, during, and after treatment. Under normal situations the treatment and how to use it depended solely on the type of cancer.  Different modes of treatment from home, at the clinic etc  (Clinic caring-non severe condition) though principled  Treatment ordering  Teaching the patient about the medicine  The process has an element of  When to start treatment  When to continue  When to stop the treatment  Dosing decision/deciding when to determine the dose  Alternating treatment when?  Instructions were given to patient during care OR policies |
| Sometimes they make a program and they call me on phone and we talk on the phone and they send transport fees and other fees to go and see the patient if the patient is very weak while going along, I go and with the medicine (take the medicine to them if they are weak so I keep going there to check on them/monitor why/ because if I keep going to monitor them especially if he/she is weak because if they are weak and then add on the burden of coming to see me, they get very very tired and you may not know if they are improving or not. why I want to find them at their home is to monitor and see if they are improving as the distance may make them tired making me fail to judge whether they are improving or not to avoid confusion **[Stefano]**  I have moved the whole country treating them. I used to go there and treat them from their homes, but I got tired, these days I tell them to bring the pts here... They used to tell me that there is a patient for treatment in such and such an area. On the patients' transport, they come and take me and bring me back here. I go with my medicine and treat them there; I go and treat them, and I come back for how long? sometimes I may stay there for a month. If it's very far I may spend a month or more treating that pt. if the pt is nearby and there is transport. What do you do, you are for me I treat your patient, and they return within a day or week. **[ROSE]** | Managing or treating patients from their home/ home caring  Monitoring the patient at home/home visiting for pt. comfort and Avoiding tiredness that may obscure the judgment of improvement  Patient calling and making an appointment (Maintaining patient comfort-outcome of care)  Sending transport fees to TMP to see the weak patient (home care)  Taking care of the weak patients at their home  Taking medicine to the weak patient | Caring for the patient at home. | Is usually conducted when the patient is in worse condition and can afford to pay the TMP. The outcome is patient improvement or reduced patient discomfort, and fatigue (improved patient comfort). Treating self from home or TMP treating the patient at their homes  This can be placed under the changing practices as an adaptation to care or stand as a 6model of care during special cases /adapting care to patient situations/conditions |
| Solome had been diagnosed with HIV and had abandoned ART for 2 years owing to the nature of her job (sex worker). She had been also diagnosed with cervical cancer, and she was in a worse condition, so I started her on my herbal medicine, and she further deteriorated. So, I stopped that treatment and decided to start her on a low dosage (herbs to increase water or blood cells, and glucose); once she improved, I started her on a full dose (herbs for cancer).  But when we went there together, after more tests, we found out that she had many other illnesses and they told me, maybe because of all those other illnesses, is the reason why she failed to improve so she gets treated now at another herbalist treatment site.  **(Faridah, 38-year-old herbalist, central Uganda)** | Observing Changing eye color-Using intuition, experience, and recognizing the signs of a worsening patient situation  Finding all possible solution  Increasing the herbal medicine dose  Not forcing treatment  Reducing dose in worse conditions  Referral to orthodox doctors for further investigations  Referral to the hospital for conventional treatment  Restarting the full dose  Resuscitating the patient or giving first aid – increasing blood cells, body fluids, and energy.  Re-tempering down  Tapering and increasing the dose in worse conditions  Tapering the herbal medicine dose | Managing a patient in advanced and worsened situations | During the care of the patient, the TMPs tried to manage soever patients they came across in the hope of giving hope to the patient while maintaining confidence in the TMP, which is key in maintaining a lasting relationship. The other outcome could be the improving patient’s health. In all cases or situations, the TMP demonstrated expertise and did something to help the patient |
| When preparing herbal medicine for cancer, I always mix in medicine for ulcers and anemia, because some people may have ulcers or anemia, which my medicines may worsen.  **(Rose, 40-year-old herbalist, western Uganda)**  I did not know about those illnesses; so for me, I was only treating cancer. No! I will of course change treatment on such occasions! Of course, I will first find out if they are HIV positive or not, if positive I ask them if they are on (ARVs) drugs. Of course, I don't have such drugs for HIV I am sure nobody has herbal medicine for HIV. So, if such a person with cancer has HIV, such a person needs lots of counseling. I first ask them if they are on any medications (ARVS) and then if yes, which medicine? Because those drugs have levels or categorizations, there are first line, 2nd treatment, etc. so I first ask her to tell me the treatment they are on and then they tell me the treatment, I then ask them when they take such medicine. Because usually, they have time, they take such medicine. If she or he tells me that they take such medicine at night, I then find out if they have TB. Because people with HIV always have issues with TB? I then get to know if they have TB or not. if they have no TB, I then treat them. **[Faridah]**  There was a man in some village (name withheld) whom I treated him he had diabetes mellitus in advanced stages but also cancer. They had found him, through investigations that he had cancer and he had medical records I used the plants I showed yesterday to treat him, and he was cured of cancer, but his blood sugar did not cure. Even today he is still there with his blood sugar (diabetes mellitus).  I give him herbal medicine for both cancer and diabetes mellitus and as well he continued taking his conventional treatment for diabetes mellitus, but when you mix the herbal medicine weakens the conventional therapy (because herbal medicine is much stronger than conventional treatment).so I said if I continue mixing them, I may cause my patient to die so I told the patient to stop the conventional treatment and I only used herbal medicine. So, we treated cancer and it was cured but he remained with diabetes mellitus today and still gets the injections. **[Rose]**  You put him on herbal medicine for cancer and then she gets drugs for hypertension elsewhere (hospital). My herbal medicine for cancer also helps cleanse the colon (overcomes abdominal distention or constipation **[Stefano]** | Concomitantly treating comorbidities and cancer  Concurrently treating cancer and comorbidities using herbs  Escorting, advising, and seeking care for comorbidities  Increasing the herbal medicine dosage /Adding more herbal medicine than previous herbalist's medicine  Inquiring about other comorbidities  Inquiring about treatment for comorbidities  Mixing herbal medicine for cancer and other comorbidities  Mixing herbal medicine for comorbidities with that cancer  Reassessing and diagnosing comorbidities  Re-assessing and diagnosing the patients -comorbidities affecting treatment efficacy  Recommending the concomitant use of herbal medicine for cancer and conventional drugs for comorbidities  Separating cancer medicine from the medicine of other comorbidities (evolving practices)  Treating after inquiring about comorbidities  Treating patients with cancer while taking drugs for other comorbidities  Treating patients with comorbidities-resulting in severe side-effects | Managing a patient with other chronic diseases | This was one of the challenges TMPs faced |
| So a patient who wants to get cured, there is no way you will reduce the dosage of medicine for them. If you reduce it, the medicine will not work for example if an orthodox doctor/ white doctor gives you coatem, he/she gives a full dosage and tells you how you are going to take it, he then tells you to go and take, so it is up to you to go and take anything either water or passion fruit juice. Likewise, also us when we give them medicine we tell them to try and go and eat, you don't eat, the medicine will not work and the disease won’t go away and the treatment will bring side effects if not eaten with food.  I gave it to one individual a female who almost died of it. You know patients have a belief that African herbal medicine has no overdose, so she took it and she almost died. So when the treatment caused the side effects she came complaining, also she had taken a long without improving, I told her to take a lot of water and stop taking medicine for about three days and continue to take it as prescribed.  Another person came saying that the medicine did not work for her (did not improve) and she had cancer of the throat. But when I asked her how you took it, she said "once she took it and started drinking it, she stopped it after thinking that it could work for a short period," she thought that it works like an injectable, once you are injected and symptoms subside, you can now give up on treatment. When I told her to continue taking it, she said that for me I am tired of herbal medicine ha! Yet she had come for more medicine that's how our patients are. The medicine burnt her throat and caused a lot of abdominal pain and it also weakened her, remember whenever somebody gets abdominal pain, the medicine would have created sores in the abdomen **[Karoli]** | Advising the patient to take plenty of water  Advising the patients to mix water, sugar, and salt in herbal medicine  Describing the side effects of handling the process  Ordering the patient to cease herbal medicine intake for a specified period  Perceiving herbal medicine to be safe  Receiving patients reporting side effects of treatment (how does he manage them?)  Referral to orthodox doctors’ resuscitation  Reporting GIT-related distress  Reporting side effects of the medicine to TMPs  Resuming intake of prescribed medicines | Managing a patient with treatment overdose | What are the key codes here? Oral route  Stop, drink, and resume treatment during side effects |
| That woman or lady, is now not with me here but she is living, she is the wife to my brother-in-law, I used to treat her, I gave her medicine for cancer for ones a year, but I realized that she was not improving, I asked her why is she not improving? What has caused that? She had cancer of the breast, so I told her to know I think my medicine has failed to help. what can we do? So you sit with her like your friend but you didn't try to push her away, then I asked her," for you in your feeling which hospital, do you think will you feel at home or at peace to get care from? Or which hospital would make your life easier/ is easy to receive treatment? Or go to? she, was able to pick the treatment center herself, surprising she again picked an herbal-related facility in Masaka herbal treatment center, where she went on to be treated or managed [Faridah]. | Advising continuous intake of HM  Believing herbal medicine has faster efficacy.  Characterizing patients who fail to improve  Disclosing treatment failure to the patient  Establishing the reason for treatment failure  Failing to complete the course of treatment  Failure to follow treatment guidelines  Getting tired of herbal medicines  Guiding the patient to find a solution  Investigating for the presence of comorbidities  Learning more about treatment failure from other TMPs  Nonadherence to treatment  Noticing/realizing treatment failure  Realizing the patient’s health was not improving  Understanding the importance of adherence to treatment (does he tell his patients)/ characterizing treatment failure | Managing a patient failing to improve | Establishing the presence of comorbidities or treatment adherence-refer or manage with herbal or discharge leading to improvement or death-overall they aim to preserve life-restoration of functional recovery  Investigating treatment failure |
| If he/she is very hostile, even if you as a traditional medicine practitioner you are not in good mood, just have to settle down/calm down your nerves and listen to that patient, so that he/she talks this and that. You ask them, what did you want me to help you with? Be calm to such and such a patient, you will be able to get the best out of them. **[Faridah]**  The second challenge we get from such patients is that though such patients get that illness, most of our patients are dirty or have poor hygiene. A patient may come to you for help, but after observing them, you say “even if he/she is sick, no no” it is too worse, it is beyond as a person.  You go on counseling them, and you tell them that such a disease requires one to be clean. You don't tell them directly, but you attach advice on the illness for example you tell them that if you are using my medicine, you need to be clean, you need to be like this and this, so at the end of the day you get surprised and you find that they have become clean/cleaned themselves. And him/her will know that they are cleaning themselves because of the other illness (cancer). You find that your advice has worked, and you have benefited from him/her, what you want. **[Faridah]**  The most important thing is to talk nicely (communicate pleasantly) with your patients so that they have nothing to hide from you  **[Sarafinah]**  For patients with cancer. I always give them a lot of time while talking to them.  even if he/she has to come for medicine, I always want to communicate with them daily, to know how they are doing so that I talk to him or her. Even if he /she does not come from very far, and I call his / his phone and it's off, I can be able to reach such a person(visit) physically. I may reach that person so that I may get to know how they are doing So that I get to know if they have taken medicine or not and why? If they used medicine correctly, bathing the medicine or washing the private parts (e.g. cervix parts), how did they use the medicine? **[Faridah ]** | Calling the patient  Communicating daily  Telling them indirectly to avoid humiliation/increase adherence  Understanding the importance of being polite/patient’s perception of care  Understanding the importance of Communicating with patients appropriately  Visiting nearby patient  Keeping In touch with the patient through constant communication/ patient consulting on phone/patient ownership-the enabling factor is the phone  **Managing an angry patient**  Pouring out their misery on you  Explaining to the patient causes of anger  Calming down the patient  Listening to the patient  Advising patients to seek alternative care options  ***Managing a patient with poor hygiene***  Giving medicine and billing the patient instead of shouting  Talking to them well/ communicating well/ good communication skills/communicating respectfully  Understanding the patient’s financial challenges  Understanding the patient’s health situations/ emotional/ psychological needs- needing comfort | Continuously communicating with respect | The best example to apply/demonstrate communication skills is through managing an angry patient and one with poor hygiene  understanding the patient and calming down  The curative TMP behavior is accepting or understanding the patient and calming down, during anger (situation) in the end the patient calms down |
| My line of treatment is counseling  A person with cancer requires a lot of counseling. Why? Because that disease scares you, if they tell you that today you have cancer, your self will start fearing. A person with cancer requires a lot of counseling like a person who has HIV.  because in most cases when we get cancer, we think that this is the end of life because we know cancer does not cure" when they say I am sick of cancer, I get anxious, a cancer patient requires a lot of counseling, he or she should not drink alcohol because of the medicine he uses.  He /she needs to take his /her medicine but as well must move on with his / her life normally. Getting cancer is not the end of the world, life must continue. We have seen so many people with cancer, who have lasted longer with it. You see they have cancer but as well they have achieved many developments or put up many developmental projects but for some, once they know they have got cancer, he/she gets depressed or loses interest, and they say why do I need to do that, after all, I have cancer, they say I will not use such and such because I have cancer, yet this is not the end of his or her life such a person; need to be counseled.  There are certain things he or she must abstain from, but you don't need to force him or her to stop those. you don't need to tell them the benefits of using that thing because everything has two sides. it has aside benefits and side effects ("bad" sometimes the patient may tell you that he/she drinks alcohol because they want to forget their problems, but I tell them that life is very important. So they may look at drinking as a way of "forgetting their worries" yet for me, I show them or am more concerned about their life **[Sarafinah].**  **But also I turn that person / such a person** into my brother/sister? Because such a person needs a lot of counseling to cope with the disease and adhere to treatment. counseling is very key in cancer pts, although we always skip it. Do you know that even if a person has no food or medication, but you counsel her, do you know that such a person will say they are better? So, I make him/her my best friend. So I give that herbal medicine  If you start using herbal medicine for cancer especially if the cancer is of the reproductive organ “owawansi" I always tell "if you have started using this medicine, delay having sexual intercourse with men for how long? maybe I tell the client to first pause a bit for a month, because of the love for life, first leave the other (sexual acts), so that we first treat the disease. But if you continue, you will find that as sores dry up(subside), because of sex you continue breeding them "okugakosa”  I told you I tell them to obtain for a period of 1 month, then take medicine daily, so I also tell you that before resuming the sexual act, first go for a medical checkup (recheck) I like it that if you take my medicine, every 1 month, go back for medical checkup, to see where we have reached (to see any improvement"  if the outcomes of the medical checkup say that the disease has not subsided "kikyaganye", even I tell you that first delay it further or first leave sex, so that we can first continue to treat you.  She used to give them herbal medicine, she used to cook herbal medicine and give it to them. But also talking to them. You know people of the village, they used to go to a secret place (banana plantation) and they would sit there and chase children away and they would finish all their issues there. They would sit there and you would think they are conversing when she is explaining how She/he should take the treatment. They used to keep their secrets, something that is not common today, but today I won't show off. We no longer even keep our patient's secrets. That's why people of long ago were better than us  **[Faridah]**  First, the basic principle is to know why somebody is sick, second, is to understand what things they have been using most. You discourage this person from using most of them. You then discourage this person from using those things (e.g. alcohol), and you tell them to use natural foods without any chemicals. Thirdly that person is given (herbal medicine) medicine remove to toxins (ebikyafu or wastes ) from the body and the blood, such a person can cure **[David]**  Yes, I tell them that cancer is an illness that doesn't cause sudden death if you get anxious, yet illness doesn't require one to be anxious a lot (kwelalikirira) you need to have hope and know that I am going to get okay, so I gave the patient hope and once they have hope, it causes one to use the medicine in time or adhere to treatment.[**Matia]**  At first, if I see they have an illness that does not cure, yet also I believe no illness does not cure, I counsel that person and tell them, my friend did you get to know that you have such an illness? If they say yes, you ask them for you how have you prepared yourself for this illness, in your heart (spirit), if they show a lot of fear, you must create conditions of support and tell them that this illness must get healed, so that they remain having hope so that they live longer and also not to discourage them from taking treatment.  After that, you ask them about what food they eat and whether they sleep well. You know if a person has such an illness, and they are not eating well (quantity and quality of food and frequency) and have poor sleep (insomnia) that illness usually worsens. The disease that would have not caused damage and would take a short period, ends up being a worse disease and the person contracts it and dies. Therefore, counseling is needed greatly where we emphasize enough resting periods, to eat well.  Why resting?  If they rest the disease does not move faster and they usually don’t get stressful thoughts. If they are moving and at times they don’t have money, sometimes they are hungry and they don’t eat in time. Stress may kill them, so you allow them to rest enough. All medicine you boil and drink or wash under the private parts. [Karoli] | Abstaining from sex  Adhering to medicine is key to improving  Adhering to treatment  Counseling/instruction depends on TMPs’ prescriptions  Counseling to take medicine  Counseling without disrespecting the patient (not screaming at the patient)  Counseling patients on the intake of natural foods  Counseling patient to Eat before medicine  Counseling patients based on the type of cancer (specifying advice to the type of cancer)  Counseling patients to consume healthy foods to increase medicine effectiveness, Side effects, and adherence  Counseling patients about the intake of healthy foods  Counseling patients on treatment  Counseling patients to adopt healthy eating habits  Counseling patients to take food before medicine -avoid S/E  Counseling sugary drinks to overcome medicine side effects (life-threatening-hypoglycemia  Counseling the patient about the intake of a healthy diet  Counseling the patient on the preparation of healthy foods  Counseling the patient on the importance of adherence to treatment (adherence counseling on subsequent visits)  Counseling the patient to adopt healthy eating habits  Counseling the patient to eat natural food or healthy food  Counseling the patient to find another treatment site  Counseling the patient to take medicine after food -general advice visav specific  Counseling on diet  Counseling/recommending adopting healthy dietary habits  Counseling/telling the patient to take a nutritious diet  Appetite  Asking about nutrient intake to avoid worsening  Asking the patient to take medicine continuously  Avoiding alcoholic drinks  Basing on the patient's condition give pieces of advice  Befriending during counseling leads to disclosing  Being calm  Blood increasing foods  Caring for the sore  Changing eating habits  communicating with respect/communicating respectfully  Continuity of caring advice or counseling via mobile phones  Continuously Talking about adherence  Counseling about positive living with cancer  Counseling according to cancer type- Counselling on delaying sexual intercourse  Counseling process  Counseling the patient  Counseling them  Creating conditions of support  Counseling depends on the conditions of the presentations  Discouraging unhealthy lifestyles  Eating organic foods  Emphasizing adherence to treatment  Emphasizing enough rest  Emphasizing the intake of nutritious foods  Emphasizing treatment adherence on subsequent visits  Emphasizing treatment adherence over the healing period  Encouraging the adoption of healthy eating habits  Encouraging the continuous intake treatment  Energy giving food  Establishing the cause of the illness (obtaining a nutritional history from the patient)  Explaining to the patient the importance of Adhering to treatment  Explaining to the patient the importance of adhering to the intake of nutritious diet-body building (vitamins/ proteins)  Fulfilling the patient's need for privacy/understanding the patient's need for confidentiality  Giving extensive counseling in presence of comorbidities  Goal: restoring hope to live longer, living longer and treatment adherence (outcome)  Having hope  Hope leads to treatment adherence  Inquiring about disease awareness  Inquiring about disease preparedness  Inquiring about having enough rest  Lengthy treating period  Long-standing asymptomatic illnesses  Maintaining/fixing the dosage during food scarcity  Medicine requires the intake of a healthy diet  Mixing herbal medicine in foods/Treating while replacing nutrients  Moving in blood  Ordering the patient to find and eat a healthy diet.  Ordering the patient to maintain a healthy lifestyle  Persuading the patient to stop alcohol  Prioritizing counseling over medicine  Reassurance about wellness  Reassuring the patient about healing  Recommending adopting healthy eating habits  Recommending diet  Recommending low salt intake/natural salts from plants (Learning from village eating habits & using them as a basis for future teachings)-the cultural basis for counseling  Recommending natural food  Recommending unfried and zero-sugar drinks, and unprocessed foods  Requiring extensive counseling  Recommending Roughages  Seeking solutions with the patient/guiding /assisting the patient make decisions (way forward/treatment center)  Soft foods  Taking crude and refined plant materials  Taking fruits and greens  Taking greens  Traditionally counseling-under banana plantation/Culturally sensitive counseling/ keeping privacy  Treating with herbs  Turning them into a friend  Understanding the effects of stress on cancer  Understanding the need to respect the patient  Understanding the patient's health and emotional situations | Providing individualized counseling | This includes the process of counseling, communications, traditional values, characteristics, or conditions under which TMPs counseled the patients, in and around treatment.  As observed, the traditional forms of counseling inspired privacy as a core principle of care  Counseling in different conditions (nutritional counseling, medications, lifestyle)  The counseling process (has dimensions of general vs specific  Communicating during counseling (principles: privacy)  Counseling process  When to start counseling  When to continue  When to stop the counseling sessions  Constituents/components of counseling rest, food |
| I tell them to go to the doctor so that they can get advice on how to control his/her blood sugars **[Stefano]**  So if she/he is weak, I may say that the patient will not tolerate (manage) the medicine. First, go to the orthodox doctors and come back again when you are much stronger, and I will start you on treatment **[Hajji Hassani]**  Yes of course, it is a patient who tells me that I have improved and then I will tell them to go back to the hospital for a checkup. When they return, I always ask them how they have progressed they in turn tell me that this and this is missing **[Jesica]**  I always tell her/him to try their level best to ask the doctors how big the sores are. If his sores are developing, how big are they? From there you understand which medicine to use **[Faridah]** | Advising the patient to Seek information from the orthodox doctor about disease severity (in case they don't escort)  Briefing TMP about outcomes of investigations on return  Directing patient for diagnostic details  Receiving referrals from Orthodox doctors  Referral to check for signs of improving health  Referral to orthodox doctors for diagnosis  Referral to orthodox doctors for further investigations  Referral to orthodox doctors for further management  Referral to orthodox doctors for resuscitation  Referral to orthodox doctors in case of suspected advanced cancer  Referral to orthodox doctors to confirm signs of improving health  Referral to orthodox doctors to manage the comorbidities  Referral to the hospital to confirm treatment outcomes/signs of improving health  Revealing self to orthodox doctors as part of learning  Sending the patient for further check-up | Referral | Referral encompassed all levels of care from diagnosis to comorbidities, to worsened patient, and checking care improvement. Whenever the TMP faced challenges beyond what he/she could handle they referred the patient to obtain the orthodox doctor's opinion before they proceeded with care. So, referral in this case is cross-cutting |
| But for me to know that they have healed they have to go back to the hospital and investigations are done to prove that they have healed. They go back to the hospital and the wound/sore is scanned or they go back to the x-ray  If a patient came when they are swollen and now, they are not and they have not died, what other evidence of healing will you need apart from that? If they not walking and now, they walk, if they were not eating and now, they eat after seeing all that evidence, does it refuse you to ask for other evidence? He or she may not have healed but when or they have improved (changed). **[James]**  The patient himself tells you. That I am now fine. (okay) for you can you undress and then see them? I don't I only receive verbal reports **[Tereza]**  There is a patient who comes for example, a patient who has cervix cancer may fail to sit like the way I am sitting now, when they sit upright, he/she is like a person who is seated on a spear. They walk bent. Now to know they have improved, you find the one whose back was bent comes now when it is not, the one who used never used to sit upright, now sit upright also when you make others your friend, he/she tends to tell you. **[Sarafinah]**  Last week I called her and asked her how she feels, and she told me that she used to have a fowl smelly discharge. For example, she would not sit with people nearby. But she told me she no longer smells for people. She also told me that she used to have pain in her back, loin, and suprapubic pain but she no longer feels it. **[Faridah]**  The patient who improves will tell you, but we also examine them to see if they have improved **[Yozefinah]**  The patient himself or herself tells me that they have healed, and if he has a wound, I observe that the wound has disappeared. I may tell the patient to go back to the hospital for a medical checkup, so the patient comes back and tells me, you see my doctor (traditional medicine practitioner) they have checked me (investigation) I am now okay. If I had the machines for investigations, I would do it but now I don't have them. I need to obtain help from white doctors. **[Hajji Hassan]**  If a patient came when they are swollen and now, they are not and they have not died, what other evidence of healing will you need apart from that? If they not walking and now, they walk, if they were not eating and now, they eat after seeing all that evidence, does it refuse you to ask for other evidence? He or she may not have healed but when or they have improved (changed). But for me to know that they have healed they have to go back to the hospital and investigations are done to prove that they have healed. They go back to the hospital and the wound/sore is scanned or they go back to the x-ray **[Silvia]** | Advising the patient to confirm progress/Frequent medical checkup/Resuming treatment after medical checkups  Ascertaining return to work/ functional recovery /  Asking about symptoms as a means to ascertain improvement  Asking them to report progress  Assessing the patient's condition on subsequent visits  Assessing the patient's condition while on treatment  Basing on signs of improving health to change the dose  Basing on the observed changes in the patient’s clinical symptoms  Basing on the orthodox doctor’s assessment to ascertain improvement in the patient’s clinical symptoms  Basing on the patient’s self-reporting of the changes in clinical symptoms  Calling and finding out the patient’s condition  Calling the patient to establish any signs of improving health  Changing medicine if not improved  Confirming disease improvement  Declaring/telling improvement  Disclosing symptoms  Examining the patient for signs of improvement  Feeling good about the patients ‘Improving health  Following up with patients to ascertain the change  Improved clinical symptoms (characterizing cancer healing)  Informing TMP about improvement/ Telephoning TMP  Judging level of improvement  Maintaining medicine dosage upon improvement  Monitoring progress is like other chronic illnesses e.g DM  Observing for clinical symptoms of improving health  Observing for symptoms of improving health  Obtaining hospital reports  Ordering the patient to report signs of improving health  Patient reporting improvement  Patients self-reporting improving health  Receiving patient self-reporting signs of improving health  Receiving patients self-reporting their improving health  Referral to check for signs of improving health  Referral to the orthodox doctor to confirm signs of improving health  Referral to orthodox doctors to confirm healing  Referral to orthodox doctors to confirm signs of improving health /regression  Referral to orthodox doctors to confirm treatment outcomes  Referral to orthodox doctors confirms the patient's improved health  Returning to pick medicine and self-reporting their improving health  Scanning and confirming cancer  Seeking information about the change in symptoms  Stopping the treatment after being sure of healing  Talking to the patient/self-reporting improvement  Telling signs of improving health  Observing the patient's condition | Confirming the patients ‘improving health | Occurred during convalesce, revisits |
| The only challenge I have now is, where I used to cut these trees (medical plants), they used to grow in a specific place where they were many, together, they have reclaimed the land and planted sugar cane there, they have removed all trees stumps [Tereza]  First of all the missionaries who came to Africa, killed you, by telling you that African traditional medicine was for satan. Today however the educated and those who are called "saved" (Pentecostals) are just starting to understand African traditional medicine. They die in ways they should not have died of. The Pentecostals say that all African traditional medicine is for satan and then you ask them to do the western medicine you take made in London? Or they grow it from the moon up for me if you bring here your savedness I chase you away **[Hajji Hassani]**  The only challenge we get from cancer patients is (entondo) pride. Second, the patient whom we bill like one million and they try hard to pay you but after treating them by the time they heal (improve), you will find that the money they gave you is over, you have even started using your money to buy medicine and treat them. They will even tell you that I have not come even with transport to take me back yet I need to fetch medicine. I have not even eaten, you can see that you get them money and give them transport money because you can't admit and feed them and also you give them medicine. So, they improve or cure or at times they don't heal. You know we treat based on two outcomes cure or death that is why they put a mortuary in Mulago, not 100% so that whenever you go to Mulago that you heal. [Karoli]  Most of them are poor and have no money, so many cannot afford to pay. You give them the medicine they cure, they go and they don’t return, they usually don’t pay and yet I have put in my money to go and fetch the medicine. People usually undermine herbal medicine, when they find you here and you ask them to give 100000/= or 60000/=, they will say all that money is for what? I have 20000/= only. Just help me, they forget that for you, you charged them only 100000/= of which you have injected a lot of money for transport to fetch the medicine, prepare the medicine, bought charcoal to make medicine so a person only wants to give 20000/= because of undermining the value of the medicine, thinking maybe the herbal medicine will not work. Sometimes they give you half pay they cure and after curing they don’t come back. So today some traditional medicine practitioners don’t bother to help because they can't afford to inject in money for free when they have a family to look after and pay rent, so that is why some traditional medicine practitioners don't focus on it. Somebody says I spend my time and money to pick medicine who is going to take it even if you know the plants, so they will reach here and start to undermine you that give me and I take and first get healed, once I am healed, I will pay you. For example, there is a DM patient we decided to give herbal medicine for free to test if it is working so she testimony for us to others if the medicine works or not, we said since you send you to have high blood sugar, let's give you medicine, so we put money together and prepare for her but when the medicine is over, she wants you to go and check on her to find out if it is over, so such a person has not behaved responsibly to ask for more treatment, yet you are treating her for free. If she would at least tell us that medicine is over and come for it, nothing so they end up saying that they are even tired of traditional medicine, yet they have improved. Bedridden one can move, and this leads to traditional medicine practitioners being powerless.  QN What determines dosage?  We tell patients these days to take four jerrycans for example instead of three jerrycans because of the scarcity of plants. In four jerrycans some plants are missing so the strength of the medicine is low. So somebody is given four jerrycans and they complain that I took four jerrycans of medicine and I did not improve because the traditional medicine practitioners made weak medicine. Because you as a traditional medicine practitioner are not instructed to work with many plants, you are tired of searching for this and this plant and adding it to the concoction. They tell you that such and such a tree is in such and such a place, you have to travel there and so it is cumbersome yet that is what makes the medicine much stronger so that you tell the patient to take a small bottle of medicine instead of only mixing 3 plants and tell a person to take a whole 20liter jerrycans of medicine and after you add them another. They always say they are tired of herbal medicine. It comes from searching or researching, the more you search for specific plants the better for patients. But you fail to go to specific places or areas to search for plants because you don't have money. After all, they will tell you that such and such a plant is in this place x and y. Yet you have to mix those trees to get concentrated juice, you then tell an individual, I am going to give you 2 bottles, if you finish them, go back to the hospital for a check-up, so that they can see your progress. So we mix many trees to get concentrated juice that is why we tell them to take little medicine.  Patients are funny, they come seeking medicine sometimes when you have it, when you ask for 50000/= they tell you that it is much and even they ask you that they don't know if it heals and would like to first test it to see if it heals, they beg you to accept 10000/= after sometimes they return and say they feel better and that you should add more medicine for 10000/= you know the economy is not good, help me, so such patients discourage us yet they come with a big bottle to take much medicine (a big bottle of two cups equating to one liter). So they finally cure at 20000/= and yet this 20000/= can't take you to and from Masaka where you got those plants that healed the patients [Jonan] | Avoiding admission and feeding costs  Charging high prices  Chasing away very inquisitive patients (undermining the TMPs)  Difficulty in accessing land with medicinal plants  Difficulty in finding herbs  Difficulty in finding medicinal plant-making care costly (adaptation)  Difficulty in finding rare species of trees  Difficulty in paying treatment fees  Prematurely discharging conditions (failure to follow TMP principles/ clinic policies)  Discharging patient  Dumping patients at TMP’s home/draining TMP’s resources  Expenses affecting care prices  Experiencing anger from patient relatives upon death  Experiencing high transport costs  Experiencing treatment fatigue  Fail to search for plants due to high transport costs  Failing to admit patients  Failing to afford free services  Failing to obtain herbs/medicine  Failing to pay for TMP’s services despite care after healing (in all situations of paying little, promising & not paying, they are still happy with their roles because of the humanistic values instilled in them)  Failure to pay treatment fees  Harvesting medicine far away  High cost of medicine  high prices of HM materials  Increased transports  Incurring costs to obtain medicine  Intake of discouraged foods or drinks  Linking traditional medicine to evil  Nonadherence to treatment  Not levied taxes  Not paying  Not paying for care  Not returning after receiving mild changes  Obtaining herbs from far  obtaining materials from far /  Obtaining medicine far  Patients take more medicine due to the scarcity of plants  Rampant deforestation  Reduced medicinal plants  Refusing to eat recommended food /  Shifting from practicing traditional medicine to other jobs.  Spending on finding medicine  Spending money to obtain herbs  Transport  Treating for free and paying later  Treating from their homes/treating as an outpatient  Treating on a part-time basis due to low profitability  Undermining the TMPs  Uprooting medicines  Waiting a long period for medicine | Barriers to patient care | Disable caring events from taking place |
| I call her / his phone [Faridah]  Some of the medicine we use to treat cancer comes from Rwanda or DRC. We just telephone them, and they deliver it, so we don’t know about the cost they incur, so after they have delivered the medicine to us, we send them their money, once we send the money, it is a must they must send us medicine although the medicine is costly, I don’t know about the taxes [Annah]. | Taking treatment as per instructions to avoid premature discharge  Importing herbal medicine  Treating for free  Treating irrespective of faith  Sourcing for patients through adverts  Telephone calling and delivering the medicine  Obtaining dosages from the internet  Feeling good/ happy when the patient improves  Informal collaboration with other TMPs or orthodox doctors | Facilitators of patient care | This enables caring events to take place |
| Our treatment aims to heal somebody, and they cure the whole body so that it can return the body to normal functioning, as God created that person **[Jonan]**  There was a patient who had cancer and when he came to me, I treated him. So, when he went back to the hospital the doctors found that the tumor alongside other smells was healed/ reduced by the herbal medicine. So, the doctors told him that the herbal medicine he was using did have a benefit on him **[Stefano]**  The patient said to me “I went to the scan, and it showed me that the liver cancer had been healed [Hajji Hassani].  What leads to a patient failing to heal is when you start them on treatment when it's too late when the cancer is so extensive when they are too weak but if they are so strong or still very strong, such people must always get healed when they are too weak, they usually die. Does not death exist? People die like elsewhere **[James]**  If we find advanced cancer, there is a likelihood of dying from cancer despite the same treatment. but if the person was early-stage cancer the person is likely to cure**.**  Dare Just give me a patient with cancer and find out if he/she doesn't heal? What leads to a patient failing to heal is when you start them on treatment when it's too late when the cancer is so extensive when they are too weak but if they are so strong or still very strong, such people must always get healed when they are too weak, they usually die. Does not death exist? People die like elsewhere **[Silvia]**  I treated him with herbal medicine. They had put him in his NGTS (pipes), I found him when he was very weak, they gave him to me and I treated him and he healed, they even paid me fully my balance, but he died after 2 years  It heals in six months (6 months).  **[Stefano]**  If the person realizes that they have cancer and starts taking food, such a person may live longer with the disease but only if they discover cancer before it has widely metastasized (before cancer has destroyed his /her widely), but for you scientist, you say cancer is on the infection (microorganism) that attaches that body part [David]  Not really, the majority are not on chemotherapy. Usually, the one on chemotherapy and my treatment will not heal [Stefano]  1) Cancer of the liver is very difficult to cure especially if the liver is enlarged  2) Lung cancer also very difficult to heal or cure  3) Throat cancer especially if the throat has already been occluded, but if he comes early, it can be cured as long as he or she can pass food through the throat and swallow.  4) Any late-stage cancer cannot cure  The problem with cancer is bringing the patient to the clinic when he/she is in an advanced stage when they have been sick for quite a long period. when he is almost done with life. When certain body parts that would keep him alive cannot keep helping him /her a patient who comes late is more likely to get treatment failure.  They are usually discharged by modern health hospitals (mainstream health care system)  Those we get early, when they have checked/investigated them, and have been told that they have cancer of the breast. But if they investigate and they tell a patient that they are going to amputate or chop off the breast, such a person their cancer is usually advanced or late-stage  **[Silvia]** | Accepting that patients fail to improve thus their death  Advanced cancer does not heal especially if they cannot eat or take treatment  Attacking TMPs after patient death  Below 70 years \  Characterizing according to treatment outcomes  Characterizing cancer according to healing time  Characterizing healing cancers  Characterizing healing wounds  Characterizing Patients by treatment outcomes  Characterizing patients by treatment response  Characterizing cancer according to  Classifying cancer according to prognosis  Curing hospital treatment  Dying  Dying (outcome of treatment failure-why because these are the first & last points of care)  Dying after healing cancer  Healing cancer but dying of other comorbidities after cancer HM treatment  Explaining the advantages of using herbal medicine  Fail to heal  Fail to heal or die (outcome)  Failing to heal when on Chemotherapy and herbal  Feeling a difference after treatment  Feeling good about the patients ‘Improving health  Gradual healing (outcome)  Healing  Early-stage cancer Healing (early-stage vs advanced)  Healing cancer and whole body  Healing completely  Healing depends on age, stage of cancer, and years of cancer  Healing depends on dietary intake  Healing faster  Healing or dying a dignified life (treatment goal)  Healing patient (the concept of healing originates from their observations  Healing slowly  Healing visav not healing  Improved patient health  Improved patient’s clinical symptoms  improving  Improving health as fast as possible  Improving on medicine  Maintaining medicine dosage upon improvement  Never experiencing unhealed patients  Nonhealing illness  Normalizing body functioning/ (Return to normal functioning)  A patient who heals cancer  Patient worsening and dying  Perceiving effects of the medicine as signs of cancer healing e.g diarrhea/vomiting (cannot respond to them or reduce dose-perceiving that as one vomits then cancer is getting out)  Performing ADLs independently  Preventing illnesses  Prolonging life  Reappearing due to nonadherence to treatment  Receiving and treating a poor patient  Receiving patients' self-reporting signs of improving health  Reduced size of the lump  Regaining the patient's health  Regressing and reappearing or regrowing  Regressing wound/tumor  Regressing/ reducing tumor  Removing cancer-causing agents leads to healing  Removing the dirt from the body  Restoration functional  Resuming economic activities  Taking long to heal for an unknown reason  Using specific plants to eliminate symptoms/ using natural painkillers  Worsening and dying when concomitantly taking herbal and chemo  Worsening symptoms before dying | Characterizing the patient’s treatment outcomes (clinic audit, after healing or death or treatment) | Healing seems to have a dimension of healing, gradual or dying but encompasses many properties such as improving clinical symptoms, size of the lump, conditions of healing, tumors or cancerous sores that healing  The outcomes of the patient's healing have an influence on the patient's Outcome (healing or not)  Characterizing patients who healed from cancer (outcome concept) |
| Yes, there are people I have ever treated, and cancer failed to heal in the hospital, after the hospital had failed, they send me that patient when they are weak. After weakening, I told them honestly that for this and this patient, I may fail to cure him or her because the cancer is advanced it is in stage (iv) so if I fail, it is not because of me but because the problem originated before admitting such a patient [Stefano]  You may be there, and they call you that your man (traditional medicine practitioner) sees this and this, but you see the man has died. He/she improved a little bit, but you doctor (traditional medicine practitioner), you are thieves, you eat his/her money now he has died.  Similarly, another relative of one of the patients who died called me instead complained, and even called me a thief and that you are a conman. So, in treating we are not conmen, there are always two outcomes either to heal or to die, even in hospital people die after spending a lot of money **[Karoli]** | Accusing TMP of consuming their funds  Attacking TMPs after patient death  Fearing a patient from dying in one's hand  Openly telling patient caretaker about prognosis before treatment, especially advanced-stage cancer  Receiving and discussing the patient’s condition during clinic visits  Telling the patient their prognosis before treatment (advanced preparing pt expectations)-opening up to patient about their status | Discussing prognosis | Pretreatment discussion of the prognosis-discussing prognosis  Characterizing or judging the prognosis & discussing it with the patients (preparing the mind & avoiding expectation.  *What do you think is the outcome (additional questions)?*  During pretreatment  Talking to the patient during clinic visits/relatives |
