# Supplementary material for: The meaning of caring for patients with cancer among traditional medicine practitioners in Uganda: A grounded theory approach
Source: PLOS Glob Public Health. 2023 Jul 17;3(7):e0001764. doi: 10.1371/journal.pgph.0001764 (PMC10351711; doi:10.1371/journal.pgph.0001764)
Supplement: S2 Table — (DOCX) [file pgph.0001764.s003.docx]

**S2 Table: Conditional Matrix Guide**

| **Category** | **What (collective definition)** | **When (during)** | **Where (in)** | **Why (because)** | **How(by)** | **Consequence (with what consequence)** |
| --- | --- | --- | --- | --- | --- | --- |
| Prioritizing life over money | Perceiving that life is much more important than money, thus the TMP was able to treat all patients regardless of their financial status. | - General patient care - Encounters with poor patient | - TMP clinic/home - Care of the patients at their homes | - Marketing/ Attracting/   it encourages more patient-to-patient referrals (marketing)   - Patients were expected to return with other illness - Recognize that some people may not afford | - Providing free care - Allowing patients to pay in phases - Charging less on treatment | Creating a long-lasting socio-economic relationship |
| Calling to serve humanity | Feeling positive towards all patients during care regardless of their conditions or presentation. | - General patient care - Care of patients with advanced-stage cancer - Wound care (fungating tumors) | - TMP clinic/home - Care of the patients at their homes | - The role of TMP is a calling to serve - Accepting that you live to serve all people regardless of their status - Fear of God’s or predecessor’s rebuke because of   Obtaining the gift for free | - Having a strong and tolerant heart or attitude during patient care/having a positive attitude towards patient care irrespective of their situation - Stronger-hearted or resilient to patient care in different conditions. - Believing/ having confidence in yourself that you have abilities to care for fungating tumors - Having mercy on the sick and being Willing to help - Treating with compassion - not feeling or showing disgust | Restoring Hope in life  Feeling comfortable (relaxed)  Increasing patient confidence in TMP  TMP being trusted by community/ community buy-in |
| Embracing traditional social values in patient care | Treating the patients as if they were a member of your community or family (related or had a blood relationship). | - General care of the patients - Handling difficult circumstances like an angry patient - Poor patients - Worse health conditions - Newly diagnosed with cancer | •TMP clinic/home  •Care of the patients at their homes | - The perception that it is a calling to serve the community - A feeling that the TMP is part of the community/ Sense of belonging - Aids in community acceptance - Settling into the role | - Treating patients with courtesy - Befriending the patient (showing them love) - Accepting and accommodating the patient - Being empathetic - Owing the patient | Providing selfless care  Providing compassionate care (part of holistic care)  Buy in the TMPs/acceptance of the community |
| Prioritizing hygiene during patient care | Ensuring proper hygiene during and after patient care as a preventive measure of cross-infections. | - During the care of the cancerous wound - After wound care - During the drying & preparation of the medicine | •TMP clinic/home  •Care of the patients at their homes | Fear of contracting cancer since it is believed to be contagious  Creates confidence in the TMPs | - Drying medicine on a tumpline - Use of gloves/polythene & discarding them away in the toilet after use) - Avoiding treating a cancerous tumor during menstrual periods - Drying clothes used during treatment under the sun | Creates confidence in the TMPs  Reducing cross-infection |
| Initiations to practice/ initiating the TMP into the ancestors’/predecessors’ practices | Ways or processes through which the TMP learned how to treat cancer. | During the pre-practice years  (Especially during childhood) | - At their predecessor’s home or family houses - Hospital | - Extending knowledge of care from one generation to another - Part of role inheritance from their predecessors’ | - Learning through Seeing/observing the TMP treating - Learning from the predecessors through seeking and providing information, involvement in care & teaching about (medicine, preparation, diagnosis, severity, etc) - Learning through the cancer disease experience - Learning through predecessors’ treating the TMP | Becoming an expert/ Starting to treat with basic expertise  Feeling confident and comfortable in-patient care |
| Inheriting my predecessor’s role | Taking over the cancer management role of their predecessors. | - Following the death of their parent or any other incapacities - Separation from the family during marriage or migration - Aging of the parent and the TMP becoming an adult. | - TMPs’ ancestral home - TMPs’ new homes - TMPs clinics | - Helps in transitioning caring from one generation to another - Preserving cultural heritage | - Receiving appointment to the role by the dead predecessor through dreams - The parent requesting their child (TMP) to take over their role | Ensuring continuity of predecessors’/in predecessor’s role.  Ensuring continuity of care |
| Evolving TMP roles | Taking over newer other roles beyond the traditional caring roles. | - In the practice years - Mass health education - During patient referrals | - The Community - In the hospitals | - Marketing purposes - Learning from orthodox doctors - Owning the patients’ care - Changing economic environment | - Escorting patients to the hospital during referrals and acting as caretakers - Communicating/ teaching masses about cancer via radios, TVs   (Mass communicator) | Maintaining the patient's comfort  Feeling comforted/a feeling of comfort in the client  Creating commitment and confidence in the TMP.  Creating a long-lasting relationship due to TMP committed  Increasing the public’s cancer awareness  Ensure a multidisciplinary approach to care |
| Maintaining their predecessors’ practices | - Practicing similar roles to those of their predecessors with no innovation. | - In the practice years - During patient care | •TMP clinic/home (especially rural TMPs)  •Care of the patients at their homes | Settled in predecessor’s role and feel comfortable  Lack of funds and technology to innovate | Harvesting, Preparing, store medicine as instructed by predecessors’  Basing/mixing herbal concoctions like their predecessors’ | Maintaining a similar quality of care as their predecessors’ |
| Evolving practices | Differences in caring practices across TMPs. | Practice years  Preparation of medicine  Care of patients | In the TMPs’ clinic  community marketing  Care of the patients at their homes | Adapting to the changing social economic environment  Competition | - Modified approaches to care - Diagnosing and managing cancer-related comorbidities (HIV, DM, HTN) - Packaging and developing standard medicinal formulas and prescriptions - Improving customer care (calling the patients, visiting, and Allowing patients to book an appointment with TMPs before visits) - Prioritizing patients over taboos - Innovative practices during adversity e.g during COVID19-delivering medicine to patients/ central points. - Increase in dishonest practices (negative)-fake products | Changing/ Improving the quality of care  Increasing accessibility to care  Improving the TMPs social economic status |
| Continuously acquiring new knowledge based on circumstances | Receiving newer knowledge about cancer and its care beyond the predecessor’s instructions. | During the practice years  Receiving challenging patient conditions/ newer cancers | •TMP clinic/home (especially modern/ urban clinics)  •Care of the patients at their homes | Expanding presentations of cancer or newer cancer that were not known to TMPs. Or were not part of their traditional understanding of cancer  Being a requirement from regulatory bodies (NDA)  Mimicking orthodox doctors | - Communicating continuously and receiving knowledge about medicines from dreams - Researching medicine/ trial and error - Reading modern textbooks about the disease - Escorting and learning from orthodox doctors | Individualizing care/ Providing on a case-by-case basis  Improving the quality of care |
| Confirming the type of cancer disease | Finding evidence to support the patient’s claims of having cancer. | The patient's first presentation to TMP's clinic for care with or without a proper diagnosis from the hospital | •TMP clinic/home | Ruling out other cancer-related illnesses.  Treating what you know  Forms basis for treatment initiation | - Obtaining history and medical records - Asking for medical records - Observing the signs of cancer - Examining the patient - Characterizing cancer according to symptoms - Differentiating cancer symptoms from other illnesses - Differentiating cancerous sores from other sores - Using experience to conclude on cancer type - Referral to orthodox doctors for diagnosis - Confirming cancer using plants | Having full knowledge of a patient's cancer disease.  Matching the cancer disease with treatment  Ensuring you are treating the right disease with the right treatment |
| Characterizing and categorizing/classifying patients with cancer | Sorting and describing patients with cancer according to certain characteristics such as severity, referral status, etc. | - Patient reception - After confirming that one has cancer | •TMP clinic/home | - Forms basis for case-by-case management/ individualized care - Helps in determining the treatment and the dosage of HM to give. - Determining and advising on prognosis - Determining which patient to admit | - Sorting patients according to disease severity (severe/advanced vs nonadvanced). - Sorting them according to means of referral (patient of patients, hospital (advanced), self-referral - Sorting according to spiritual problems & assistance with other issues vs cancer alone | Enrolling patients into care/determining whom to admit or not  Ensuring appropriate and individualized care |
| Preparing medicine for the patient | Obtaining and preparing medicine for the patient. | Initiation of treatment and follow-up/ subsequent visits | - TMP clinic/home - Community (herbs) | The inability of some patients to prepare the medicine.  Avoiding cancer-related stigma in the community if seen preparing medicine | - Collect herbs - Prepare - Boil-single/Multiple - Pack - Store for emergency | Ensuring the right formulation  Ensuring the patient’s convenience/convenient for the patient to take the medicine  Ensuring continuity of care |
| Individualizing care | Varying treatment decisions according to the patient's condition and the TMP assessment. | Initiation of treatment and follow-up/ subsequent visits | - TMP clinic/home - Community (herbs) | The expectation that the patient would heal | - Varying treatment types, dosages, and time depending on the patient's conditions - Varying treatment initiation practices - Varying treatment by symptoms or cancer type | Provide/ensure appropriate treatment |
| Continuously communicating with respect | Talking to patients while respecting them regardless of their social or financial status. | Patient care (initiation and follow-up)  Counseling  During poor hygiene and interface with an angry patient | At TMP’s clinic/ home  At patient home | Vital to Keeping a lasting relationship  Ease in Influencing behavior change8 | Understanding the patient's financial situation  Understanding the importance of proper communications  Constantly communicating with the patient via face-to-face and phone  Not shouting at patients  Indirect communication (link problems/patient weakness/ poor hygiene to wellness or effectiveness of treatment) | Building a strong continuous caring relationship  Creating a lasting relationship  Owning the patient/confidence in the TMP  Calming down the patient  developing a sense of belonging  Feeling being comforted |
| Providing client-centered counseling  Providing individualized counseling | Clients are guided, given options based on identified problems during care, and are allowed to choose what they can manage. | During the care of patients especially at   1. First contact 2. Diagnosis with comorbidities 3. Initiation of treatment 4. Follow up 5. Concomitant use of herbal and chemotherapy 6. Depending on certain cancers 7. Treatment failures | At TMP’s clinic/ home  At patient home | Restoring hope and positive  Encourage treatment adherence  Avert social stigma  A belief that cancer requires extensive counseling | Following counseling principles and techniques while talking to the patients such as befriending the patient, persuading the patient, allowing the patient to choose  Discussing with patients issues that are pertinent to their health such as nutrition, sex, rest, diet, lifestyle, adherence  Providing general counseling | Restoring hope and positive living  Influencing behavior change  Relieving anxiety |
| Referral for further consultation and investigations | Telling or sending the patient to the hospital or other TMPs in face of patient challenges or ambiguities. | Worsened patient conditions  Presentation to TMP without diagnostic records  During treatment/ improvement | At TMP’s clinic/ home  At patient home | Due to a lack of technological abilities  Confirm diagnosis Resituate the patient  Further management of cancer or comorbidities  Confirm treatment success or improvement | Telling the patient to go to the hospital  Giving patients instruction on what to obtain from doctors (diagnostic details,  Telling the patient to check for improvement | Form basis for further management/ helps in further management decision making |
| Confirming the patients ‘improving health | Seeking information about the change in the patient's condition following the initiation of treatment. | Convalescence  Having taken treatment for a long time  Patient complaints | At TMP’s clinic/ home  At patient home | helps in further management decision making  feeling good if there are positive signs of improvement-proof that their treatment is working | Asking, calling, and patient telling signs of improving health  Examining the patient  Referral of the patient to the hospital | Helps/aids in further management decision making  Acts as a basis for managing another patient |
| Barriers to patient care | These are factors or issues that limited access to care. | The caring process | At TMP’s clinic/ home  Community | Because they indirectly limited care by increasing the cost of care | Increasing the cost of transport to obtain medicine (deforestation)  Raising the cost of care in case of Dumping the patient at TMP's home and high taxes  Lack of funds to advertise | Limiting access to care |
| Facilitators of patient care | These are factors or issues that enhanced access to care | The caring process | At TMP’s clinic/ home  Community | Because they indirectly limited care by reducing the cost of care or increasing access | Providing free or cheap care or allowing to pay in phases  Importing and delivery of medicine  Calling and delivering medicine (technological advancement)  Treating patients irrespective of their faith  Informal collaboration | Increased access to care |
| Patient’s treatment outcomes (clinic audit, after healing or death or treatment) | The TMP describing what happens after his/her treatment intervention. | During treatment  Convalescence  During treatment failure  After patient demise | At TMP’s clinic/ home  At the patient's home via phone | Signifies treatment effectiveness  Determining the patient’s prognosis | By assessing/describing various possible outcomes. Described differently by different TMPs   1. Dying/failing to heal/worsening   Visav  Healing/improving/gradual healing/ healing the whole body while replacing nutrients   1. Reduced lump/tumor/regressing mass visav not 2. Restoration of body functions/performing ADLs independently/ normalizing body functioning visav not 3. Prolonging life visav not     Finding and providing reasons for each outcome  Healing vs dying | Informing future treatment practices  Guides future treatment experiences-who and when the TMP will put future patients that present with similar conditions on treatment/ who and who not to admit in future  Helps in determining prognosis in the future |
| Discussing patient prognosis | The TMP discussing treatment outcomes with their patients. | Throughout the whole care  Initiation of care (advanced cancer)  Convalesce | At TMP’s clinic/ home  At the patient's home via phone | Preparing the patient's mind about treatment effectiveness (managing expectations)  Averting patient’s caretakers’ hostility in case of death/ unexpected outcomes | Assessing and classifying patients by prognosis  Telling the patient  Providing positive reassurance (adherence can improve the patient's condition) | Reducing TMP blame  Influencing behavior change (those that expect to cure)  Relieves anxiety  Restoring the patient's hope/  Removing false hope from the patient and family.  Psychologically prepares the patient for any eventualities.  Promotes end-of-life care |
| Managing a patient failing to improve | These are steps taken after several attempts to treat a patient with no improvement. | During patient treatment failure | At TMP’s clinic/ home  At patient home | Seek further management.  Rule out other diseases  Basing on humanistic values  Seek expert opinions  Helps in discharge | Realizing not improving patient  Discussing and guiding the patient to the solution  Investigating the cause of improvement or comorbidities  Referral | Ensure the patient obtains appropriate care or expert opinion  Ensures collaborative management  Avoiding unnecessary death |
| Managing a patient with treatment overdose | Managing the side effects of the TMPs' treatment. | During patient overdose | At TMP’s clinic/ home  At patient home | Reduce/stop further complications, morbidity, and mortality  Preventing death | Reporting  Ordering to stop  Restarting treatment later after receiving proper instructions  Teaching about the correct use of medicine  Referral | Restoring the patient's general health  Prevent body damage |
| Managing a patient with other chronic diseases | Caring for the patient irrespective of the number of diseases they present with. | During a presentation with comorbidities, | At TMP’s clinic/ home  At patient home | Enabling faster healing or enabling quick healing  Reducing complications of a particular disease | Inquiring about comorbidities  Assessing for them  Re-assessing and diagnosing the patients -comorbidities  Giving HM for comorbidities  Referral & escorting | Restoring  the patient’s hope in life |
| Managing a patient in advanced and worsened situations | Caring for patients with late stages of cancer often after discharge from the hospital. | During a presentation with advanced-stage cancer | At TMP’s clinic/ home  At patient home | Believing that they have a solution to every illness  believed that herbal medicine was superior to western medicine | Using intuition, experience, and recognizing  Tapering the dose  Increasing the dose  Finding solution  Referral  Resuscitating the patient | Restoring hope in life  Prolonging the patient’s life  Improving patient’s conditions |
| Home-based caring  Caring for the patient at their home/in-home care | The TMP takes care of or manages the patients from their homes. | During severe illness (advanced cancer) | In the patient’s home | Make proper judgment of the patient's improvement  Prevent patient fatigue  Customizing or individualizing care | Paying for TMP transport and upkeep  Contacting the TMP via phone call  Observing or assessing patient care  Taking treatment with them | Promoting the patient’s comfort  Customizing or individualizing care  Reduced patient fatigue  Preventing further complications  Aiding proper assessment outcomes |
