# Supplementary material for: The meaning of caring for patients with cancer among traditional medicine practitioners in Uganda: A grounded theory approach
Source: PLOS Glob Public Health. 2023 Jul 17;3(7):e0001764. doi: 10.1371/journal.pgph.0001764 (PMC10351711; doi:10.1371/journal.pgph.0001764)
Supplement: S3 Table — (DOCX) [file pgph.0001764.s004.docx]

**S3 Table: Reflective coding matrix**

|  |  | **Reflective coding matrix** | | | |
| --- | --- | --- | --- | --- | --- |
| **Core category** |  | **Restoring patients’ hope in life through individualizing care** | | | |
| **Processes** (actions/interactions) | Ensuring continuity of the predecessor's role/in the predecessors' role | Having full knowledge of a patient’s cancer disease | Restoring hope in life | Customizing or individualizing care | Improving patients’ conditions/health |
| **Properties** (characteristics of the category) | Becoming an expert | Matching the cancer disease with treatment | Psychologically preparing the patient for any eventualities | Providing better quality care | Characterizing the patient’s treatment outcomes |
| **Dimensions** (location of the property on a continuum) | - Initiating into predecessors' practices - Calling to serve humanity - Embracing traditional social values in patient care - Providing compassionate care (part of holistic care) - Inheriting my predecessor’s role - Maintaining a similar standard of care as their predecessors - Continuously acquiring new knowledge based on circumstances - Evolving practices - Modified approaches to care - Evolving TMP roles - Increasing the public’s cancer awareness - Changing/varying standard of care | - Confirming the type of cancer disease - Characterizing and categorizing patients with cancer | - Providing individualized counseling - Maintaining the patient’s comfort - Feeling comforted - Ensuring the patient’s convenience/convenient for the patient to take the medicine - Continuously communicating with respect - Discussing patient prognosis - Promoting end-of-life care - Calming the patient - Developing a sense of belonging - Owning the patient - Creating confidence in the TMPs - Creating commitment and confidence in the TMP | - Individualizing care - Prioritizing life over money - Increasing accessibility/access to care - Preparing medicine for the patient - Reducing cross-infection/ prioritizing hygiene during patient care - Referral/ensure collaborative management/ ensure a multidisciplinary approach to care - Reduced patient fatigue/caring for the patient at their home - Preventing further complications - Ensuring the right formulation - Managing a patient in advanced and worsened situations - Managing a patient with other chronic diseases - Managing herbal medicine overdose - Managing a patient failing to improve - Barriers to patient care - Facilitators of patient care | - Confirming the patient’s improving health - Healing/improving/ gradual healing/healing the whole body while replacing nutrients - Reduced lump/tumor/regressing mass - Restoration of body functions/performing activities of daily living independently/ - normalizing body functioning - Prolonging life - Dying/failure to heal/worsening (variations) |
| **Contexts** | Feeling confident and comfortable in-patient care | Ensuring appropriate dosing of treatment | Relieving anxiety  Reducing TMP blame | Obtain appropriate care  Informing future treatment practices  Acts as a basis for managing another patient | Determining prognosis in the future |
| **Modes of understanding the consequences** (outcomes of the process) | Buy in for TMPs/acceptance of the community  Provide selfless care | Enroll patients in care/ determine who to admit | Influence behavior change  Improve adherence to the treatment protocol | Prolong the patient’s life  Avoid  unnecessary death | Create a strong, long-lasting/continuous caring relationship |

Note. The table categories and subcategories were created from the consequences, major categories, and some codes or concepts in the conditional matrix guide.
