# Supplementary material for: The meaning of caring for patients with cancer among traditional medicine practitioners in Uganda: A grounded theory approach
Source: PLOS Glob Public Health. 2023 Jul 17;3(7):e0001764. doi: 10.1371/journal.pgph.0001764 (PMC10351711; doi:10.1371/journal.pgph.0001764)
Supplement: Attachment — Submitted filename: Response to reviewer comments GT FINAL1.docx [file pgph.0001764.s005.docx]

| Comment | Response | Page/line number |
| --- | --- | --- |
| **Reviewer 1** |  |  |
| This paper provides very interesting research work addressing the Meaning of caring for patients with cancer among traditional medicine practitioners in Uganda: A grounded theory approach | Thank you very much for the compliment |  |
| 1. The introduction requires substantial improvement. | Thank you very much for the comment. In its current form, we avoided over-discussion of literature in the introduction. Instead, the introduction was used to explain the study problem, significance, scope, or study gaps that compelled us to conduct the study as recommended by Strauss and Corbin (Kenny and Fourie, 2015). We reserved an extensive literature review of the emerging theory, in the discussion. We also believe that extending literature in the introduction may not add much value.  Nevertheless, some literature has been added to the introduction. | Page 4, LN 102-105 |
| 2. Can you change the title of your paper? | Thank you very much for the comment. Given that the primary goal of an inductive qualitative research is to explore "a phenomenon of interest", we aimed to understand the "TMP's meaning of caring for people with cancer” or solely their perceptions of care. Because of that, we find it difficult to change the topic. However, we welcome any suggestions. |  |
| 3. I would suggest including more literature if possible. | Thank you very much for the comment. Relevant literature review has been added especially in the introduction and discussion section especially as specifically suggested by reviewer 2. | Page 4, LN 102-105, 642-670, 760 |
| 4. Is there any other theory of the research framework used in your study? | Thank you very much for the comment. It is possible to use a theoretical model especially when using constructivist grounded theory (GT) research (<https://doi.org/10.46743/2160-3715/2014.1014>)  Constructivist (GT) uses constructivism and symbolic interactionism philosophy, in which the researcher (interviewer) and the participants (interviewee) are co-creators and composers of the research story/theory, which is not the case with our study. Thus, the use of a theoretical framework would be more applicable in the Constructivist (GT).  We used the modified Straussian GT and were not guided by any prior theory because we wanted to develop the model inductively from participants’ data or meanings only (emic perspectives), but not deductively (etic perspectives). We believed that TMPs create their reality through interactions, and it is this reality that we wanted to put to light.  Our aim therefore was to understand their meaning of care and develop a model (from the participant or interviewee perspectives only) and compare it with existing literature to confirm or disconfirm it as recommended by Kenny and Fourie, (2015). Nevertheless, future researchers in traditional medicine may use our model as a steppingstone to develop new substantive or grand theories.  *The term “inductive” has been added to the design section in the methodology.* | Page 5, LN 113 |
| 5.  Can you include gender aspects in your study? | Thank you very much for the comment. The question is quite general, but this is our take. It may be difficult to include such gender aspects, especially in this study where concepts emerged naturally from the data (inductive). Unfortunately, gender did not emerge naturally.  The term “inductive” has been added to the design section in the methodology, as stated previously |  |
| 6. Is there any other limitations of your study? | Thank you very much for the comment. The main Limitation of this study has been added to this paper including the qualitative nature of the study that does not allow for generalization. Above all care provided by TMPs is culturally specific or unique, making generalization to another cultural group challenging. The following statement has been added to the strength and limitation section.  *“Because culture is unique and dynamic and that this study took a solely qualitative nature, generalization of the study findings to some cultures in Africa may be challenging."* | Page 28, LN 769-771 |
| 7. What is the strength of your study? | Thank you very much for the comment. One of the key strengths of this study is that this is one of the first studies to explore TMPs’ perceptions of cultural care in sub-Saharan Africa and develop a substantive theory using the GT approach to better conceptualize it. We believe this study will serve as a guide to other researchers in SSA to explore further the topic using the same methodology. As well the model can be used to conduct future research in the subject area.  The following statement has *been* added to the strength and limitation section of the paper. Details are also explained in the conclusion  *This theory may guide future research in similar cultural settings where TMPs care for patients with chronic illnesses such as cancer.* | Page 28, LN 768, Page 29, 793-800 |
| 8. How your paper is more relevant to public health aspects? | Thank you very much for the comment. TMPs are a community structure that contributes to increased access to primary health care in the community, particularly for patients with chronic conditions such as cancer in low resource limited setting like in Africa (contribute towards health equity). Patients with cancer visit them. Why patients abandon modern health care services and visit TMPs or why patients continue to use the TMPs services alongside modern health care services is a public health question. As well the quality-of-care TMPs provide is a public health concern in Africa. Given that no single healthcare system is sufficient to solve all the world health problems the WHO and nations in sub-Saharan Africa are increasingly recognizing and integrating TMPs into mainstream health care. Therefore, we must understand deeply the nature and quality of care provided by TMPs as this influences proper integration of TMPs in modern health care.  A statement has been added to the introduction to clarify the public health aspects “*Although TMPs provide increased access to primary health care in sub–Saharan Africa and countries are* *increasingly recognizing and integrating them into mainstream biomedical care, the nature and quality of care provided to patients with chronic conditions such as cancer remain elusive and a public health concern in Africa.”* | Page 4, LN102-105 |
| 10. Future recommendation: Can you try a mixed-method approach for future study? | Thank you very much for the suggestion. This has been positively taken up and a statement has been added to the conclusion and reads: *Future studies may also approach the subject of care in traditional medicine using the mixed methods grounded theory which would allow generation and testing of the GT concurrently as well as linking theory to practice.* | Page 29, LN 798-800 |
| 11. Discussion section needs minor improvement. | Thank you very much for the comment. The discussion has been extensively Improved in many aspects as suggested by the second reviewer. |  |
| **Reviewer 2** |  |  |
| Reviewer #2: This research into the Meaning of caring for patients with cancer among traditional medicine practitioners  in Uganda: A grounded theory approach, is insightful and well grounded. It is potentially a good paper that contributes original knowledge to an otherwise under-researched area, particularly with regard to social scientific studies into cancer care in low-resource settings.  However, while I really enjoyed reading your paper, I feel that the article could do with improved organization of material, tightening up and emphasis on key areas, and getting rid of repetitive material, especially towards the end of the paper. I will now detail what critical revisions are necessary to improve the overall quality of this paper: | Thank you very much for the commendation. |  |
| **Specific comments in the document** |  |  |
| **INTRODUCTION** |  |  |
| 1. ORGANISATION: the paragraph starting on Line 95 should be moved up either at the beginning of the introduction or immediately after paragraph one in the introduction. This is simply to enhance the coherence of this section. Please see more on organization at the end of this review. | Thank you very much for the compliment |  |
| **Methods** |  |  |
| 2. METHODOLOGY: While I appreciate the authors nicely explaining the GT approach and how it has been applied to data collection and analysis, this section needs further work to tighten it up and make it much more accessible to non-expert readers. I suggest eliminating the tables and either, integrating the text into the body of the work, or using simple text boxes to highlight important coding principles, while not clogging up the boxes to the extent that they are difficult to follow as is currently the case. | Thank you very much for your compliment and suggestions. One weakness of GT research is "academic elitism", which makes simplifying GT concepts rather difficult. Above all there are also arguments that oversimplification of GT concepts would not appeal to a technical reader, who may even question the validity of the study findings or may find it difficult to differentiate it from a qualitative descriptive study or other qualitative designs or hinder replication of the study. Thus, we needed to balance between the technical aspects for the scientific community and legibility for other research consumers or communities. Nevertheless, your suggestions were considered as:  We eliminated the tables and integrated the text into the body of the work by providing at least one example. However, reference to supplementary tables (1,2,3) were maintained for those who may need the details. Importantly, because the reflective coding matrix is similar to Fig 2 and at the same time it is explained under the storyline, there was no example given, but the reference to the supplementary table 3 was maintained for those who need the details. | Page 9, LN 214,222; Page 10, LN 247, Page 12 LN 288-295 |
| Secondly, I am not convinced that TMCs in this study were always able to distinguish between cancer symptoms and other illnesses because a) the authors confirm that TMCs have no formal education | Thank you very much for the comment. We accept that the education level is key to the proper identification and management of cancer. However, most traditional healers are initiated in their practices in various ways including through apprenticeship, experiential learning, etc., all of which do not require formal training. TMPs may not be formally educated but still have valuable information about cancer and its symptom. Because the information they have is passed on from their predecessors and has been learned experientially over so many years. That is why we needed TMPs with a 10-year of experience, for more credible information. Above all, previous studies have shown that informants with lower levels of education have more relevant knowledge of traditional medicine than the more educated ones. (<https://doi.org/10.1155/2022/1645265>).  Second, although not reported in this study because of the limited study scope, we asked participants (TMPs) before the interview to detail the signs and symptoms of the cancers they treated. Most had good knowledge of the major cancers they managed, which is in line with the biomedical or clinical literature, save for a few TMPs (n=2) who mixed up some cancers and other conditions. The following statement has been added to the inclusion criteria  *“TMPs had to possess relevant knowledge about cardinal signs and symptoms of at least one cancer they managed to be interviewed.”* | Page 5, 134-135 |
| and b) it is not clear whether or not they only deal with medical referrals or any other suspected cases of cancer. I suggest reframing these claims to something along the lines of ' TMCs evaluated symptoms based on own knowledge and experience and had xyz criteria for arriving at a cancer diagnosis in the absence of prior medical records...' This is because, in my own experience researching indigenous healing systems, TMCs always engage in trial-and-error practices and often rely on a repertoire of historical illness symbols to assign symptoms to non-specific categories of illness. For example, when researching fever and febrile illness in Tanzania, I found that clusters of symptoms categorized as fever included at least five out of 8 well-known categories that were passed on through generations. Additionally, treatment was not always strictly traditional, rather, some form of hybrid between allopathic and herbal medicine, and, as you rightly point out, suggestions for diet and lifestyle changes on the part of the patient and his or her carers. I would like to see these nuances brought out more in your analysis so that it does not look like TMC cancer care is a linear and homogenous practice across the districts. I am sure you would agree that this is not the case.  I suggest expanding finding number 2 to incorporate a section on what happens when patients do not have medical records confirming cancer. What would the TMC typically do? How would they arrive at a diagnosis? I am aware that in low-resource settings the vast majority of cancer cases are either never diagnosed or diagnosed at very late stages and therefore often there is no cure. It will benefit the paper to talk briefly about this. | Thank you very much for the comment. We do agree with your comments.  It is very true that some TMPs at times used traditional ways based on their knowledge and experience to confirm cancer, especially in situations where patients could not afford to obtain medical records. However due to abstraction and avoidance of the significant negatives working against the emerging theory this content had been left out. Consequently, it is also true that TMPs also dealt with both patient self-referral and medical referrals. Because of that, the following statement has been added to the theoretical concept of “Having full knowledge of a patient’s cancer disease”  “For most TMPs, those were the most important (or only) ways they used to confirm a cancer diagnosis. However, on occasions where patients (self-referral) could not afford to obtain a formal diagnosis because of the inaccessibility of cancer diagnostic services or lack of funds or in the absence of prior medical records, one TMP based on his knowledge and experience, observed patients for signs and symptoms of cancer and used specific plants and animal products (e.g., bile duct) to purportedly confirm cancer.  *In the beginning, if my patient chews or swallows my medicine (bile juice or certain plants), if the person has cancer, signs will appear (like throat pain for esophageal cancer). (Rose, 40-year-old herbalist, western Uganda)*  *This added data has also been discussed in the discussion section.* | Page 17, LN 431-439; Page 25, LN 682-686 |
| 3: ANALYSIS (MINOR CHANGES)  LINES 536-540 seem contradictory. You state that TMCs in your study believed that herbal remedies were superior to allopathic treatments, yet they send patients they couldn't cure back to allopathic doctors. Why was that? This needs a few lines to clarify and supported by vignettes. See similar contradictions on the page that follows this (Lines 553-557). Also please substantiate Lines 602 onwards. How did TMCs know that tumors were reducing if by their own admission they said they couldn’t check these with 'machines'? I suggest rewording with something less grand... | Thank you very much for those observations. It is ironic but true that most TMPs believed that their medicine was superior to modern medicine, yet they referred some patients to orthodox doctors in some circumstances such as cases where the patient had comorbidity they could not manage or some advanced cancer cases for fear of repercussions in cases when the patient died at their homes. To avoid this significant negative working against the emerging theory, we have eliminated the statement “*TMPs believed herbal medicine was superior to Western medicine”*  In addition, although TMPs did not have modern diagnostic techniques to confirm that the patients were improving or even measuring the tumor sizes, they based on their own experience and observation. Therefore, the statements have been reworded include the “how”  *“Based on their experience, on observing that their patients were not improving”*  *Based on the TMPs treatment experience and observation, Other common outcomes were included.* | Page 21, LN 563  Page 23, LN 612 |
| **Discussion** |  |  |
| 4. DISCUSSION Lines 613-723 reads like a literature review and is mostly repetitive of your findings. I suggest reworking this section, bearing in mind the original research questions and whether or not you feel you have answered them, and or how your proposed substantive theory has been applied to the findings, and the contribution this makes to existing literature (weaving in the literature review as you sometimes do). | Thank you very much for the comment.  It is important to note that after the development of the substantive theory, it would have been relevant to compare our study findings with other similar models or theories developed elsewhere (especially in sub-Saharan Africa) in traditional medicine as recommended by (Birks et al., 2009). However, most studies are phenomenological in nature and presented from the researcher and TMP perspectives.  Because of that limitation, the study findings are largely discussed using extant and collateral literature from indigenous and conventional medicine around our major categories or concepts to validate or refute our model. In addition, there is a dearth of literature on some theoretical concepts.  Nevertheless, extensive revisions have been made   1) The following statement has been inserted at the start of the discussion.   “*The study findings are largely discussed using extant and collateral literature from indigenous and conventional medicine around our major categories or concepts to validate or refute our model, because of limited literature around similar practice models in sub-Saharan Africa*.”   2) Removed repetitions/ summarized findings / reduced extensive explanation of study findings/study findings have been paraphrased and unnecessary details removed. 3) The discussion has been presented in such a way that it shows the achievement of study objectives. 4) Under contribution differences between our findings and other studies and implications/recommendations of the study findings to practice have been emphasized and immersed around the study findings. 5) The implication and recommendation section has been eliminated | Page 23-28  Page 23, 628-631 |
| 5. GENERAL COMMENTS:  Lines 725-727 should move to the literature review and then referred to back in the implications. Often, it is a distraction to include new literature at the end of a paper.  Lines 733-735 requires support with references, and so do lines 744 to 748.  Lines 761-766 is a key discussion point and should come in the DISCUSSION section and preceded in the literature review. I.e a small section detailing how TMCs training differs from orthodox doctors in Uganda, and the implication of this for managing chronic illness such as cancer in rural under-resourced settings. | Thank you very much for the observation.  All suggested sentences were returned to the discussion section.  References were added to suggested sentences. The implication section has been merged with the discussion and the conclusions  The following statement about training has been added  *Currently, the training of general medical practitioners in Uganda still largely emphasizes the medical model and not holistic and culturally sensitive care (bio-psycho-social care) which are relevant to the modern oncological approach to the management of patients with cancer [39, 40].* | Page 264, LN 653-656 |
| In STUDY LIMITATIONS, Lines 772 -774 need a brief substantiation. Are you suggesting that newer entrants into traditional healing praxtices are going in it for money? Is this a sense you got from your research or your own views? It would be important to know what makes you feel this is the case. | Thank you very much for the observation. I indeed meant new entrants into traditional healing based in towns, who may not be traditionally trained but rather sell traditional medicine products, on a commercial basis. Although this is not included in this study due to the GT nature of this research(abstraction), TMPs in this study criticized such new entrants as quacks who are only interested in money and exploiting their clients.  In addition, some may not be locally trained but traditionally trained from other countries or cultures, and thus the care they provide and principles they use may differ from those locally trained. For example, those trained in Asian countries.  Therefore, these clarifications have been made as follows  “*The findings may not fully represent new entrants who may not be traditionally trained but provide care based on modern capitalistic or business values or those traditionally trained elsewhere (abroad) and may blend traditional and foreign cultural values and principles in the care of patients with cancer”* | Page 28, 771-776 |
| **CONCLUSIONS** |  |  |
| Finally, the conclusion should typically talk about how you have answered your research question/s, what this means for further research, and how your proposed theory can further be applied to similar studies moving forward. At present your conclusion is but a summary of your findings. | Thank you very much for your suggestions. This has been revised to include   1) Whether the research question was answered 2) Suggestions for further research 3) Application of the model in other studies   Most were scattered elsewhere in the discussion; these have been reorganized. | Page 39-30 |
